# Genetic Adaptation to Growth Under Laboratory Conditions in *Escherichia coli* and *Salmonella enterica*

**DOI:** 10.3389/fmicb.2018.00756

**Published:** 2018-04-26

**Authors:** Anna Knöppel, Michael Knopp, Lisa M. Albrecht, Erik Lundin, Ulrika Lustig, Joakim Näsvall, Dan I. Andersson

**Affiliations:** Department of Medical Biochemistry and Microbiology, Uppsala University, Uppsala, Sweden

**Keywords:** experimental evolution, adaptation, growth medium, *Salmonella enterica*, *Escherichia coli*, fitness, competition experiment

## Abstract

Experimental evolution under controlled laboratory conditions is becoming increasingly important to address various evolutionary questions, including, for example, the dynamics and mechanisms of genetic adaptation to different growth and stress conditions. In such experiments, mutations typically appear that increase the fitness under the conditions tested (medium adaptation), but that are not necessarily of interest for the specific research question. Here, we have identified mutations that appeared during serial passage of *E. coli* and *S. enterica* in four different and commonly used laboratory media and measured the relative competitive fitness and maximum growth rate of 111 genetically re-constituted strains, carrying different single and multiple mutations. Little overlap was found between the mutations that were selected in the two species and the different media, implying that adaptation occurs via different genetic pathways. Furthermore, we show that commonly occurring adaptive mutations can generate undesired genetic variation in a population and reduce the accuracy of competition experiments. However, by introducing media adaptation mutations with large effects into the parental strain that was used for the evolution experiment, the variation (standard deviation) was decreased 10-fold, and it was possible to measure fitness differences between two competitors as small as |*s*| < 0.001.

## Introduction

With recent advances in sequencing technologies, microbiologists are increasingly using the power of experimental evolution followed by whole genome sequencing (WGS) as a tool to address many questions in evolutionary biology. This has led to insights in, for example, phenotypic innovation (Blount et al., 2008), evolution of antibiotic resistance (Gullberg et al., 2011; Toprak et al., 2012; Miller et al., 2013; Knopp and Andersson, 2015), convergent and parallel evolution (Tenaillon et al., 2012, 2016; Lang et al., 2013), the cost of generalism (Kvitek and Sherlock, 2013), industrial innovations (Blaby et al., 2012), and adaptation to stress (Zorraquino et al., 2017).

If the research question entails letting the bacteria evolve to solve a “problem”, for example to compensate the fitness cost of a certain mutation or evolve resistance in response to an antibiotic, a common complication of the analysis is the selection for general adaptation to the growth conditions alongside the desired specific adaptations. Not only could this phenomenon complicate the interpretation of genotype—phenotype causality, necessitating tedious strain reconstructions, but also introduce a significant risk that medium-adapted mutants outcompete the strains that adapt to the condition of interest, particularly if selection for the latter is not very strong.

Medium adaptation in the model bacterium *Escherichia coli* has been studied for certain media (Herring et al., 2006; Conrad et al., 2009; Aguilar et al., 2012; Blaby et al., 2012; Le Gac et al., 2013), and one landmark study is the long-term evolution experiment by Lenski and co-workers (Tenaillon et al., 2016) where 12 lineages of *E. coli* B have been serially passaged in minimal medium with low glucose levels for more than 69,000 generations [http://myxo.css.msu.edu/ (Tenaillon et al., 2016)]. During the course of evolution, these bacteria have, for example, evolved aerobic citrate utilization (Blount et al., 2008). However, to the best of our knowledge, no studies of medium adaptations in the well-established model bacterium *Salmonella enterica* together with systematic comparisons of different media have been made. *E. coli* and *S. enterica* diverged some 120–160 million years ago (Winfield and Groisman, 2004), and still show large similarities in their genome size (about 5 Mbp), gene content (~3,000 shared genes; McClelland et al., 2000) and metabolic capacity. However, some separating and species-defining attributes exists, which contribute to their different lifestyles. The original *E. coli* K12 isolate was a human gut commensal that contained a conjugative plasmid (F) and an active prophage (λ). However, when MG1655 was constructed in order to make K12 more amenable for genetic studies, F and λ were removed by treatment with DNA-damaging agents (Blattner et al., 1997). In addition, MG1655 contains many different transposable elements, some of which are very active. In contrast, the *S. enterica* LT2 isolate was an invasive pathogen. The lab strain, which has not been intentionally modified, still carries a large (94 kb) virulence plasmid as well as several pathogenicity islands, four functional prophages, and six functional but largely inactive transposable elements (Lam and Roth, 1983; McClelland et al., 2000).

In this study, we examined and compared mutations selected during serial passage for up to 1,000 generations for *S. enterica* LT2 and *E. coli* K12 in four commonly used laboratory media. A total of 185 different genetic alterations were identified with little overlap in mutation targets between the two species in the same medium. We systematically made genetic reconstructions of a large collection of these mutations (111 re-constituted strains with single and multiple mutations) and compared relative exponential growth rates of all of them. Additionally, we analyzed the competitive fitness of a majority of the reconstructed strains. This generated a list of mutations that are commonly selected in standard laboratory media and a set of mutant strains that are useful as pre-adapted ancestral strains for evolution experiments and fitness assays. In addition, for a subset of the mutations, we have analyzed factors that influence selection and examined the role of large-benefit mutations as source of noise in competition assays.

## Results and discussion

### Adaptation to laboratory media

**Figure 1:**
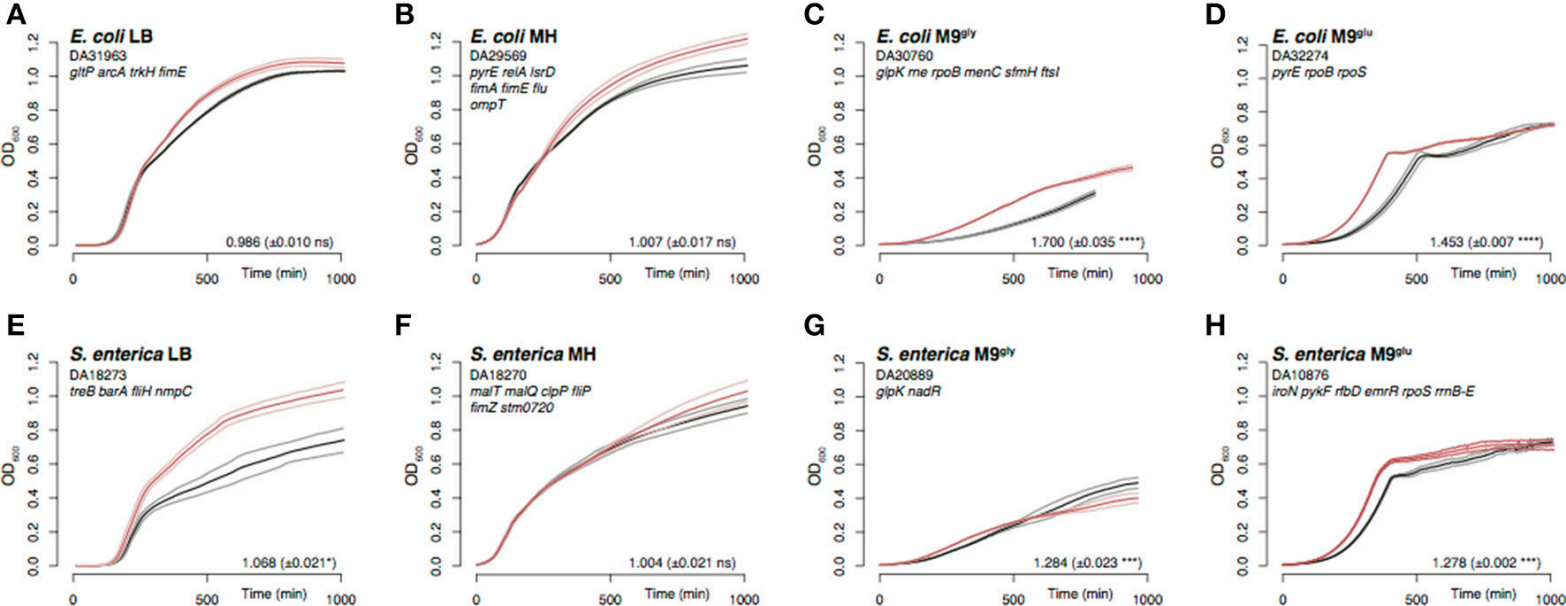
Representative examples of growth curves and exponential growth measurements for the evolved populations. Growth was monitored by OD_600_ measurements for evolved populations (red) and un-evolved wild-type control strains (black). In order to adjust for the effects of different starting ODs on the apparent lag time, the time point where the culture OD reached 0.006 was set to T = 0 min. Note that this removes any real differences in lag time. The thick lines are the averages of 2–3 replicates, and the thinner lines are the standard deviations. **(A–D)** *E. coli* evolved in LB, MH, M9^gly^, and M9^glu^. **(E–H)** *S. enterica* evolved in the same media. Genes found to be mutated are indicated for each population where those present in >70% of the reads are typed in black and those present in 10–70% are typed in gray. The relative exponential growth (± SD) is indicated for each population, with asterisks indicating significant difference (^*^p < 0.05; ^***^p < 0.001, ^****^p < 0.0001; two-tailed Student's *t*-test, equal variance) as calculated between mutant and wild-type. See Table S2 for exact mutations and Figures S1–S5 for OD_600_ and exponential growth rate measurements of all evolved populations.

To select for adaptive mutations to four commonly used laboratory media, 4–10 parallel cultures of *E. coli* and *S. enterica* were serially passaged for 500–1,000 generations in liquid broth (by daily serial passages with 1,000-fold dilutions, i.e., approximately 10 generations per day; Knöppel et al., 2017). The media selected were two different complex media, lysogeny broth (LB) and Mueller Hinton broth (MH), as well as M9 minimal medium supplemented with either 0.2% glycerol (M9^gly^) or 0.2% glucose (M9^glu^). The bacterial physiology changed during the evolution experiment, and the evolved populations showed signatures of adaptation (visualized in growth curves; Figure 1 and Figures S1–S5). Populations adapted to minimal medium had evolved faster exponential growth rates (Figures 1C,D,G,H), whereas populations adapted to complex media were primarily affected during later phases of the growth curve (Figures 1A,B,E,F).

### Common adaptive pathways

**Figure 2:**
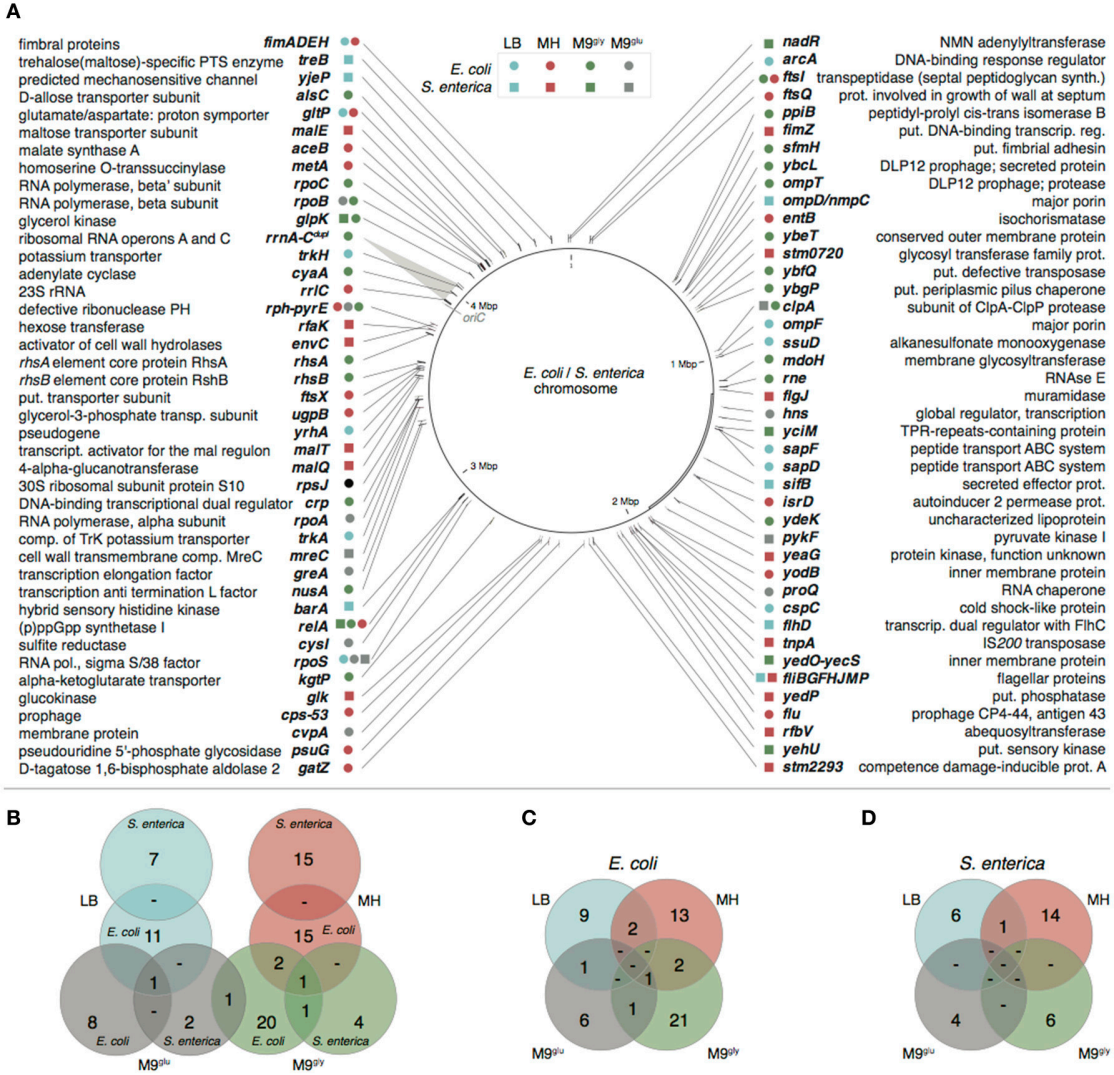
Mutated genes or loci found in the whole genome sequenced evolved lineages. **(A)** Mutational targets in *E. coli* are marked with circles and in *S. enterica* with squares. Turquoise, LB; red, MH; gray, M9^glu^ green, M9^gly^. A duplicated area spanning *rrnA*–*rrnC* is marked with a gray transparent triangle and the location of *oriC* is indicated in gray. Note that for comparison the genes were all marked on the chromosomal map of *E. coli*. The *S. enterica* chromosome differs by a large inversion as compared to this genome (marked with a double line in the figure). **(B)** Overlap of mutations between species. **(C,D)** Overlap of mutations found in *E. coli* and *S. enterica* (**C,D**, respectively).

By whole genome sequencing of the evolved bacteria we identified in total 138 independent genetic changes in *E. coli* and 83 in *S. enterica* (Figure 2, Table S1 and Supplementary Table S1 in Knöppel et al., 2017). The number of mutations in each evolved lineage varied between 1 and 11. For both species, the most common mutations were amino acid substitutions (58/138 and 41/83 for *E. coli* and *S. enterica*, respectively) but a wide variety of other mutations were also found (Table S1). Transposition of insertion sequence (IS) elements were frequent in *E. coli* (22/138) but absent in *S. enterica*. Similar to previous studies (Lande, 1983; Notley-McRobb and Ferenci, 1999; Herring et al., 2006; Conrad et al., 2010; Wang et al., 2010; Puentes-Téllez et al., 2013; Saxer et al., 2014), many of the mutations identified in our study could be putatively grouped into those that either are expected to directly affect the utilization of the resources present in the media (e.g., *glk, glpK, gltP, mal, ompD*/*F, pykF, rph, sapD*/*F, ssuD, treB*, and *trkH*/*A*), or, those that confer more global effects on gene expression (e.g., *arcA, clpA, cyaA, nusA, proQ, relA, rne, rpoA/B/C/S*, and *rrlC*).

The overlap between mutation targets in the two different species evolved in the same growth medium was small (Figure 2B and Table S1); only four overlapping genes were found (*clpA, glpK, relA*, and *rpoS*). In addition, the genes *fimA, fimE, ftsI, gltP, relA, rph*-*pyrE, rpoA, rpoB, rpoS*, and flagellar genes were mutated under multiple growth conditions in the same species. Although described as very rare (Orr, 2005; Dettman et al., 2012; Tenaillon et al., 2012), some of these genes also showed parallelism down to the nucleotide level, for example six mutations found under different conditions (*treB*[insertion of A or T at nt 541], *gltP*[A-115T], *glpK*[Val8Phe or Ile], *rpoB*[His526Tyr], *pyrE*[G-41ΔG], and *rph*-*pyrE*[Δ82 bp]). Even between the two species, examples of mutations affecting the same nucleotide (*glpK*[Val8Phe] in *E. coli* and *glpK*[Val8Ile] in *S. enterica*) or adjacent nucleotides in the same codon (*glpK*[Arg34Ser] in *E. coli, glpK*[Arg34His] in *S. enterica*) were found. It is worth noting that despite all lineages originating from single colonies, we cannot exclude the possibility that some of these mutants were already present in the frozen stocks of the ancestral strains, or that some of the apparent nucleotide level parallelism could be due to carryover between lineages during the evolution experiments. Additional examples of parallelism (also at the nucleotide level) were found when comparing our datasets with previous experimental evolution studies (Table S3). In contrast, we did not find mutations in *argR, mrdA, rho*, or *spoT* that have been commonly encountered in *E. coli* evolved under similar conditions (Herring et al., 2006; Barrick et al., 2009; Conrad et al., 2011; Tenaillon et al., 2012; Le Gac et al., 2013), indicating that growth conditions and strain background, even within the same bacterial species, can strongly affect which mutations are selected.

### Re-constructed mutants and analyses of their fitness

**Figure 3:**
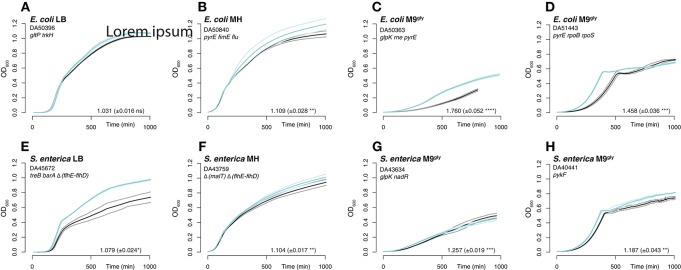
Growth curves and exponential growth measurements for re-constructed strains. Similar to Figure 1. The re-constructed mutants with the highest measured relative fitness in each media are shown in turquoise and un-evolved wild-type control strains in black. The thick lines are the averages of 2–4 cultures, and the thinner lines the standard deviations.

**Figure 4:**
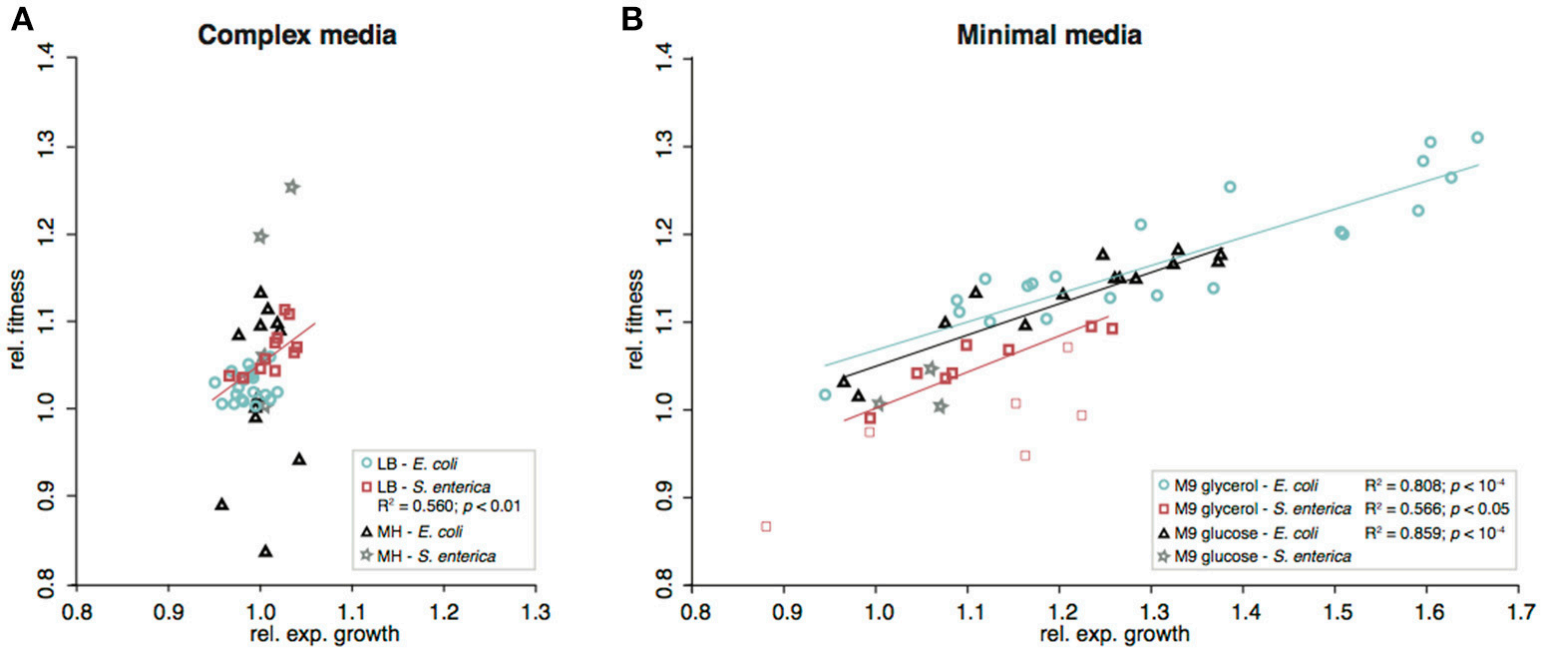
Correlation between fitness and relative exponential growth rates of re-constituted mutants. **(A)** Complex media, **(B)** Minimal media. In the cases where significant correlations (Pearson correlation) were detected, these are indicated as lines in the figure. For *S. enterica* evolved in M9^gly^, the constructed *relA* mutants showed variable results and were excluded from the calculations (included in **B** as faint red squares).

**Figure 5:**
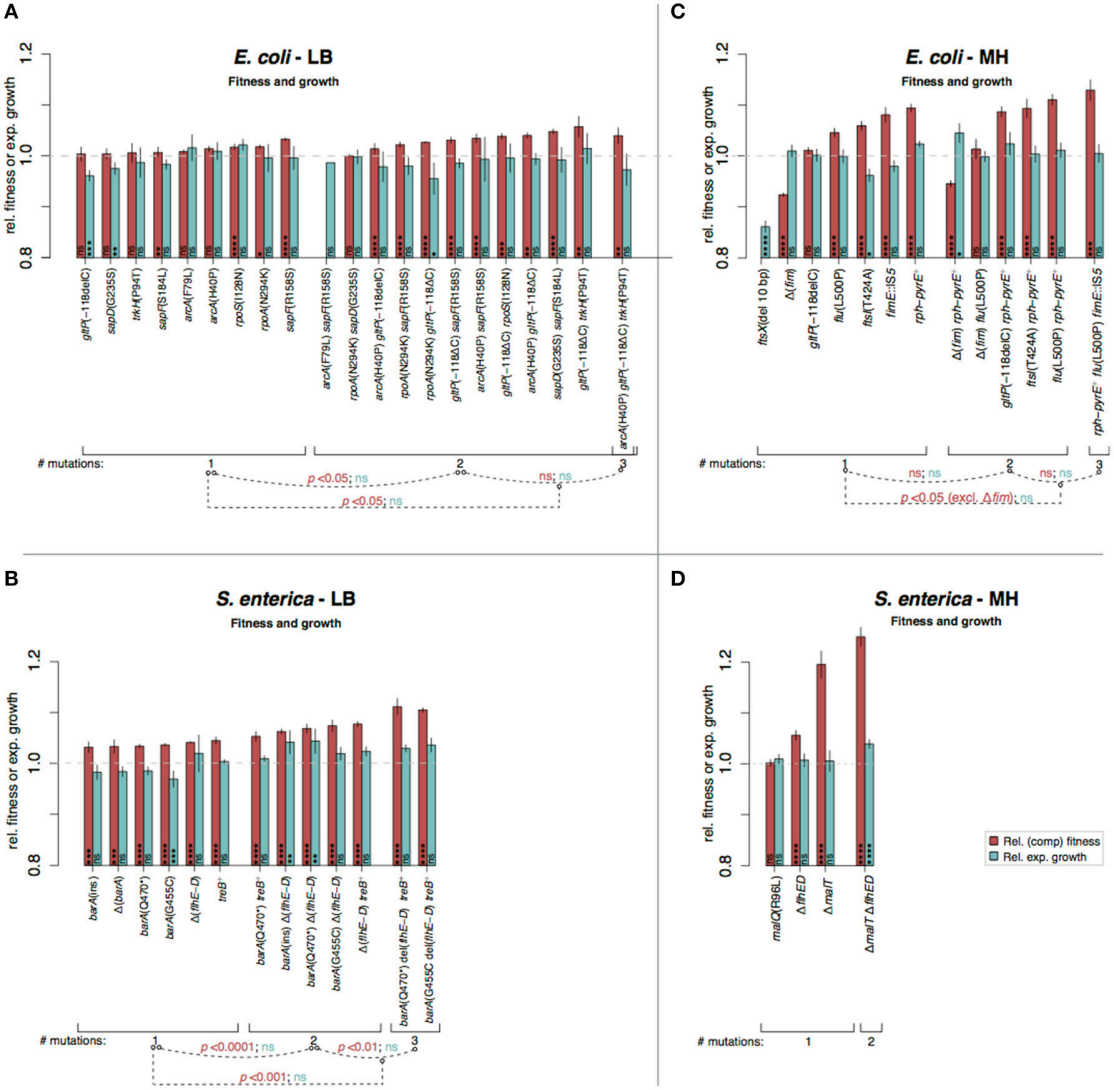
Fitness and growth measurements in complex media of re-constituted mutants. Red bars indicate relative competitive fitness (±SD) and turquoise bars indicate relative maximum exponential growth rates (±SD). The mutants are grouped according to number of introduced mutations and *p*-values indicate significant fitness or growth rate differences between the different groups (^*^p < 0.05; ^**^p < 0.01; ^***^p < 0.001, ^****^p < 0.0001; two-tailed Student's *t*-test, equal variance). **(A)** *E. coli* in LB, **(B)** *S. enterica* in LB, **(C)** *E. coli* in MH, and **(D)** *S. enterica* in MH. High variation in fitness measurements depending on medium batches was observed for MH assays (Figure S10). The Δ*fim* mutation was constructed and tested based on the faulty assumption that the *fim* mutations caused loss of fimbriae (see Supplementary discussion).

**Figure 6:**
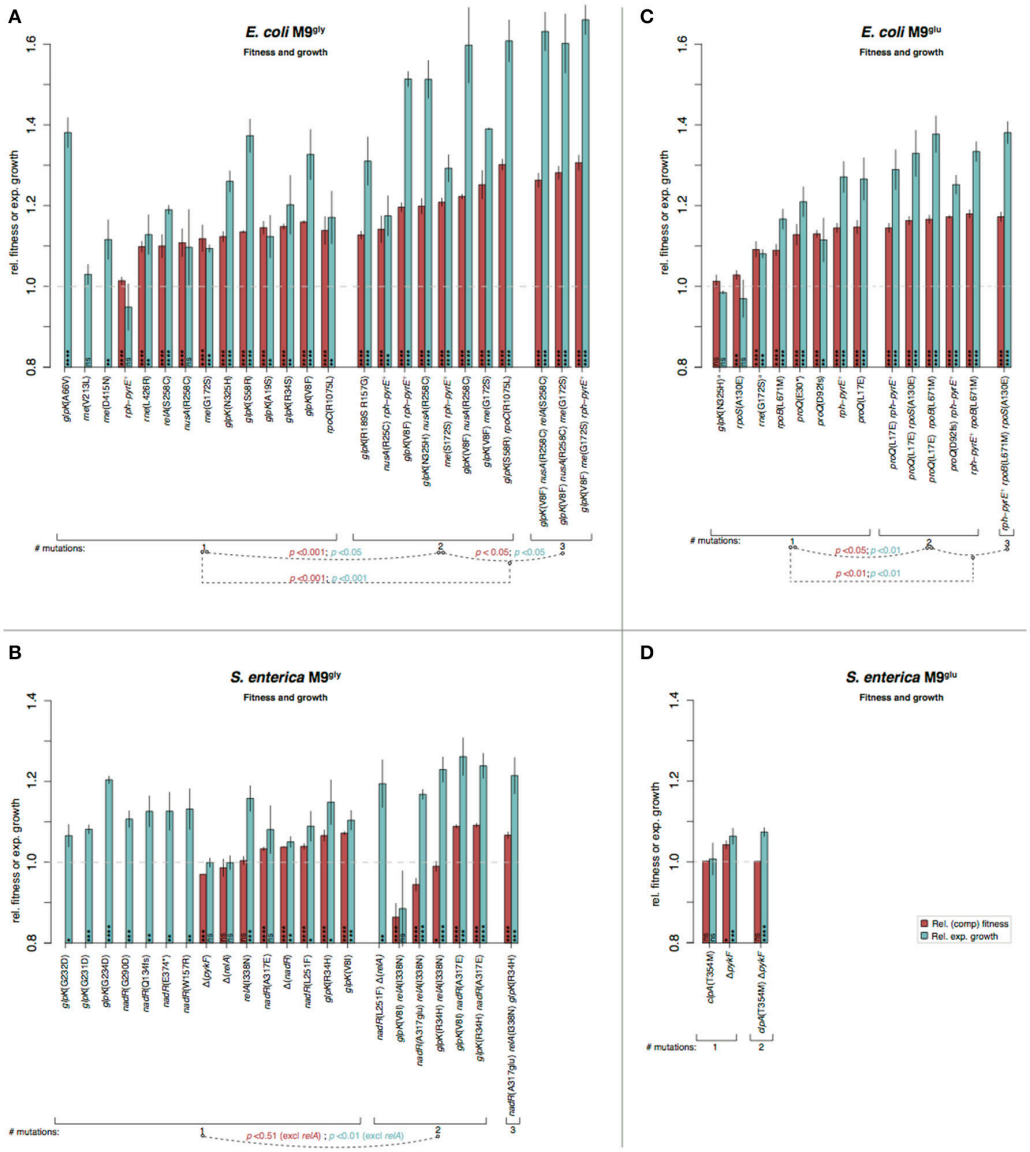
Fitness and growth measurements in minimal media of re-constituted mutants. Similar to Figure 5. **(A)** *E. coli* in M9^gly^, **(B)** *S. enterica* in M9^gly^, **(C)** *E. coli* in M9^glu^, and **(D)** *S. enterica* in M9^glu^. ^a^ Mutations selected in M9^gly^ but tested also in M9^glu^.

Further analysis of the mutants primarily focused on mutational targets that appeared in more than one population. These mutations were re-constituted in a scar-free manner (no other changes introduced than the specific mutations examined; Näsvall et al., 2017) in the parental non-evolved background, and they were in most cases combined with other mutations. Thus, the genetic compositions of the reconstructed strains resembled those of the mutants found in the serial passage experiments (Table S4). In five cases (indicated in Table S4) we tested combinations of genes that were not found mutated in the same populations. The re-constructed mutants were analyzed by head-to-head competitions (which involved the whole growth cycle: lag, exponential, and stationary phases) against the parental strain. Additionally, their exponential growth rates were determined when grown in separate cultures. In general, the mutations found in the evolved strains increased fitness when tested after re-constitution and were therefore likely to have been selected rather than accidentally fixed during serial passage (Figures 3–6). In some cases, our competition experiments did not show any selective advantage for single mutants on their own, whereas a combination of two mutations revealed an increase in fitness (e.g., *gltP*[-118ΔC] and *arcA*[F79L]; Figure 5), indicating that these mutations also had been selected but only in combination with other mutations. The mean selective advantage of all analyzed single and multiple mutants was 1.06 and 1.11, respectively. For *E. coli* grown in LB with a large effective population size, the mean selective advantage of single beneficial mutations has earlier been measured to 1.023 (Perfeito et al., 2007), and this number corresponds well to our measurement (|*s*| = 1.012 ± 0.009).

**Figure 7:**
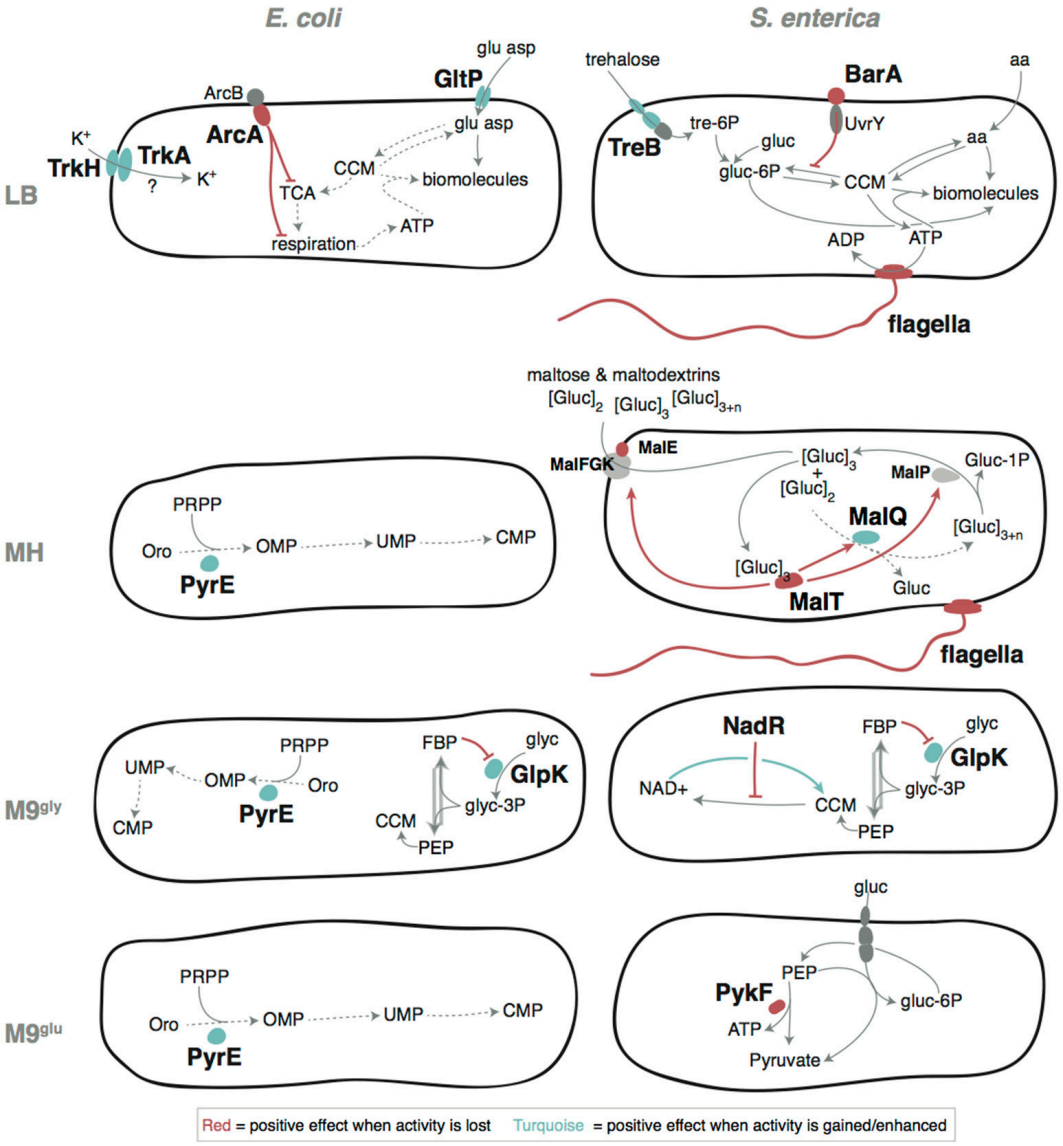
Putative adaptive mechanisms. Gray arrows indicate reactions and dashed arrows illustrate reactions that work poorly in the un-evolved ancestral strains. Colored arrows or lines shows regulatory pathways where red indicates a positive effect when an activity was lost or reduced, and turquoise indicates a positive effect when an activity was increased. tre-6P, trehalose-6-phosphate; gluc, glucose; gluc-6P, glucose-6-phosphate; CCM, Central Carbon Metabolism; aa, amino acid; ATP, adenosine triphosphate; ADP, adenosine diphosphate; TCA cycle, Tricarboxylic Acid Cycle; glu, glutamic acid; asp, aspartic acid; [gluc]_2_, maltose; [gluc]_3_, maltotriose; [gluc]_>3_, longer maltodextrins; gluc-1P, glucose-1-phosphate; Oro, orotate; PRPP, phosphoribosyl pyrophosphate; OMP, orotidine 5′-monophosphate; UMP, uridine 5′-monophosphate; CMP, cytidine 5′-monophosphate; FBP, fructose-1,6-bisphosphate; NAD+, nicotinamide adenine dinucleotide; glyc, glycerol; glyc-3P, glycerol-3-phosphate; PEP, phosphoenol pyruvate. See Supplementary Discussion for more detailed explanations of the probable positive mechanisms.

Stepwise increases in competitive fitness and in relative exponential growth for the re-constructed single, double, and triple mutants were seen for *S. enterica* in LB and for *E. coli* in M9^gly^ (Figures 5, 6). A compilation of possible beneficial effects of some of the mutations are presented in Figure 7 and in Table 1, and are also further discussed in the Supplementary Discussion.

**Table 1:** Possible mechanisms for medium adaptations.

**Condition^a^**	**Mutated gene**	**Protein function**	**Type of mutation^a^**	**Probable selective effect**	**Experimentally tested in this study**	**References**
*Eco* LB	*arcA*	Part of a two component regulatory system with ArcB. Represses genes in TCA cycle and aerobic respiration, and acts as a switch to turn on anaerobic respiration	aa substitutions, probably decreased affinity to ArcB	In combination with an up-regulation of GltP it may increase the pathways (TCA^a^ cycle and aerobic respiration) needed to convert e.g., glutamic acid into CO2 and energy. Increased metabolism of abundant amino acid	Figure 5A	Gunsalus and Park, 1994; Saxer et al., 2014
*Eco* LB	*gltP*	Glutamate/aspartate:proton symporter	Promoter mutations, increased expression	Increased uptake of glutamate and aspartate to use as carbon and/or energy source	Figure 5A, Figures S7B, S8A	
*Eco* LB	*trkH/trkA*	Potassium transporter proteins	aa substitutions	Increased uptake of K^+^	Figure S7B	
*Sal* LB	*barA*	Part of a two component regulatory system with UvrY. Activates expression of metabolic genes	Probable decrease or loss of function	Locks the system in the gluconeogenic state which is expected to be beneficial in medium lacking glucose	Figure S7A	Suzuki et al., 2002; Pernestig et al., 2003
*Sal* LB	*treB*	Fused trehalose(maltose)-specific PTS enzyme: IIB component/IIC component	Pseudogene reactivation	Improved utilization of the trehalose present in the yeast extract	Figures S7A,D, S8B, S12	
*Sal* LB, *Sal* MH	flagellar genes	Locomotion	Loss of function	Flagella and chemotaxis are not needed in cultures with rapid mixing	Figure S9	Edwards et al., 2002; Koskiniemi et al., 2012
*Sal* MH	*malT*	DNA-binding transcriptional activator for the *mal*-regulon and maltotriose-ATP-binding protein	Loss of function	Loss of the activator of the mal-regulon. Probably reduces the cost of expression of non-functional mal genes	Table S5, Figures S7C, S8C	
*Sal* MH	*malQ*	4-alpha-glucanotransferase (amylomaltase)	Compensates for a mutation present in the ancestor	Pseudo-reversion of the *malQ*(Leu96Arg) mutation which leads to a Mal^+^ phenotype	Table S5, Figure S7C	
*Eco* MH, *Eco* M9^gly^, *Eco* M9^glu^	*rph-pyrE*	Defective ribonuclease PH - orotate phosphoribosyltransferase	Increased *pyrE* expression	Relieve a pyrimidine biosynthesis defect in MG1655		Conrad et al., 2009
*Eco* M9^gly^	*rhs*	“Recombination hotspot” locus	Activation of contact dependent inhibition system	Inhibition of neighbor cells		Koskiniemi et al., 2013
*Sal* M9^gly^, *Eco* M9^gly^	*glpK*	Glycerol kinase	The mutations around aa 230 abolish binding of FBP^a^ and the mutations around aa 66 disrupts tetramerization	Increases enzymatic activity through loss of allosteric inhibition		Liu et al., 1994; Bystrom et al., 1999; Applebee et al., 2011
*Sal* M9^gly^	*nadR*	Bifunctional DNA-binding transcriptional repressor/NMN^a^ adenylyltransferase	Probable decrease or loss of function	Relieved repression of genes involved in de-novo NAD^+^ synthesis		Zhu and Roth, 1991; Grose et al., 2005
*Sal* M9^glu^	*pykF*	Pyruvate kinase I	Loss of function	Higher levels of phosphoenolpyruvate increases glucose uptake through PTS		Woods et al., 2006
*Eco* LB, *Eco* M9^glu^, *Sal* M9^glu^	*rpoS*	RNA polymerase sigma S/38 factor	Reduced function	Reduced RpoS affinity to the RNA-polymerase, beneficial under constant conditions with little stress by decreasing the competition between RpoS and Sigma 70		Ferenci, 2005; Conrad et al., 2009; Maharjan et al., 2012; Saxer et al., 2014

a *Eco, E. coli; Sal, S. enterica; aa, amino acid; fs, frameshift; wt, wild-type; FBP, fructose-1,6-bisphosphate; PTS, phosphotransferase system; TCA cycle, citric acid cycle; NMN, nicotinamide-nucleotide*.

#### Differences in fitness effects in complex and minimal media

The fitness effects differed between complex and minimal media where mutations selected in minimal media generally had larger and more variable effects than those selected in complex media (Figure S6). In LB, mutations isolated in *S. enterica* had larger beneficial effects than those isolated in *E. coli* (competitive fitness for single mutations, |*s*|^*S*. *enterica*^ = 1.037 and |*s*|^*E*. *coli*^ = 1.012; *t*-test: *p* < 0.0001), whereas the opposite was seen for mutants isolated in M9^gly^ (|*s*|^*S*. *enterica*^ = 1.026 and |*s*|^*E*. *coli*^ = 1.117; *t*-test: *p* < 0.0001).

The effects on competitive fitness and relative exponential growth rate of mutations found in the complex media also differed from those found in the minimal media (Figures 3–6 and Figures S1–S4). In complex media, the main fitness-increasing effects of the mutations were not expressed during exponential growth but rather in other parts of the growth cycle such as lag phase or stationary phase, similarly to the evolved populations. In minimal media on the other hand, the primary effects were observed in exponential growth and hence, the measurements of relative exponential growth rate and competitive fitness correlated strongly for minimal media but not for complex media (Figure 4). Conceivably, the likely explanation for this media-difference is that both organisms are quite well adapted to exponential growth in complex media, and that their exponential growth rates in those media cannot easily be improved further by mutation. In contrast, in minimal media, growth rate is limited not only by the quality and uptake of the external resources present in the medium (i.e., the sources of carbon, nitrogen, sulfur, phosphorous, etc.) but also by the “internal wiring” of the cell (e.g., regulation of metabolic pathways and trade-offs between carbon and energy metabolism). As exponential growth rate in minimal media is so readily improved by mutations, it appears that this “internal wiring” in both organisms studied here is not optimized for growth in minimal media.

#### Epistasis

Instances of positive (synergistic) epistasis, where the fitness effects of the combined mutations were larger than the sum of the effects of the single mutations, were found in 9% of the re-constituted strains. Negative (antagonistic) epistasis, where the effects of the combined mutations were smaller than the sum of the effects of the single mutations, was found in 34% of the reconstructed mutants carrying multiple mutations (Table S4). The remaining 57% could not be distinguished from additivity with the resolution of the fitness assays. The positive epistasis combinations were all found between mutational targets found in the same evolving strain. Some of the reconstructed mutations (e.g., *arcA, gltP*, and *pyrE* in some media) showed a significant increase in fitness only when combined with other mutations which indicates the most likely order in which the mutations occurred. Other mutations did not show fitness increases when re-constructed and tested (e.g., the *relA* mutations found in *S. enterica* lineages adapted to M9 glycerol, *clpA* in an *S. enterica* lineage adapted to M9 glucose, and *sapD* found in an LB adapted *E. coli* lineage). It is plausible that these mutations conferred a benefit only against other mutants that were present in the same culture at the time when they appeared, although we have not tested this idea. Other possibilities are that the mutations confer fitness effects that are below the limit of detection in our fitness assays or simply that they hitchhiked with a beneficial mutation present in the same clone.

### Adaptation to Lysogeny Broth (LB) medium

Lysogeny Broth (LB) is composed of tryptone (a pancreatic hydrolysate of caseine from bovine milk), yeast extract (from autolyzed *Saccharomyces cerevisiae* cells), and NaCl. It has been argued that growth of *E. coli* in LB stops when all available carbon sources are depleted, that LB contains no fermentable sugars, and that amino acids are the primary sources of carbon utilizable by *E. coli* during growth in LB (Sezonov et al., 2007). However, significant amounts of glucose, galactose, and trehalose have been reported, with the latter being the most abundant (Ferreira et al., 1997; Hanko and Rohrer, 2000, 2004; Baev et al., 2006). Thus, a conceivable adaptation to growth in LB would be through mutations that improve uptake and / or metabolism of available amino acids or sugars. In accordance, 7 of the 11 mutated genes in *E. coli* included different uptake systems (*gltP, trkA*/*H, sapF*/*D, ompF*, and *ssuD*) where e.g., *gltP* promoter mutations improved the uptake of glutamic acid, making it utilizable as carbon source (Figure 7). In *S. enterica*, mutations that repaired a defect in TreB, a component of the trehalose uptake system, were common, as were mutations that affect motility (Table S1 and Figure 7). Experimental tests to determine the beneficial effects were performed for *gltP, arcA, treB*, and *barA*. This, and the likely cause for loss of flagella in *S. enterica* are further discussed in the Supplementary Discussion (Figures S7–S9).

### Adaptation to Muller Hinton (MH) medium

MH is composed of beef extract, casein hydrolysate, and soluble starch, and is widely used for antimicrobial susceptibility testing. To our knowledge, no detailed study of bacterial physiology in this medium has been performed. *E. coli* and *S. enterica* cannot utilize starch as carbon source (Gutnick et al., 1969; Wandersman et al., 1979). Starch is stable during autoclaving (Hickman et al., 2009) but is degraded to simpler sugars by amylases that are present in meat (Skrede, 1983). Nevertheless, the fact that all *Salmonella* lineages grown in MH acquired different mutations affecting *mal* genes, involved in metabolism of starch degradation products (maltose and maltodextrins), suggest that significant amounts of starch decomposes to simpler oligosaccharides during preparation of MH. Interestingly, both loss of function mutations in *malT* and a mutation that repaired a preexisting loss of function mutation in *malQ* was found in the evolved lineages. Both of these mutations conferred a fitness advantage compared to the ancestral strain, which may reflect a cost of expression of the *mal* regulon in a mutant that is unable to metabolize maltose, and this cost can be ameliorated either by preventing expression of the *mal* regulon, or by restoring the function of the defective MalQ enzyme (See Figure 7 and Supplementary Discussion and Table S5). During our fitness measurements we encountered large variation of the same strain assayed on different occasions in different medium batches, possibly reflecting differences in starch degradation as discussed above (Figure S10).

### Adaptation to M9 glycerol medium

M9 medium is an inorganic sodium phosphate buffer (phosphorus source) supplemented with ammonium chloride (nitrogen source), magnesium sulfate (magnesium and sulfur source), and calcium chloride (calcium source). Essential trace elements are omitted from the recipe but are present at sufficient amounts as contaminations in the other medium components. Typically, the carbon source is the only organic compound present to serve as starting point for building all the molecules the cell needs. For both *E. coli* (K12) and *Salmonella* this represents a condition quite far from the nutrient rich animal gut where they are most commonly encountered, which may cause selection to alter regulatory networks that have evolved in other conditions. One such example is *glpK* mutations that were repeatedly found in both species and allows for better utilization of glycerol as sole carbon and energy source (Herring et al., 2006; Applebee et al., 2011). Along this line, the re-constituted mutant with the highest increase in fitness was found in this medium; the triple *E. coli* mutant *glpK*(Val8Phe) *rne*(Gly172Ser) *rph*-*pyrE*^+^ had a competitive fitness of 1.31 and grew 66% faster than the ancestral parent in the exponential phase (Figure 6A).

### Adaptation to M9 glucose medium

M9^glu^ medium has a similar composition to M9^gly^ medium and differs only in the carbon source. However, glucose is a better carbon source than glycerol, allowing for faster growth. Except for *rpoS* mutations that are commonly encountered in both complex and minimal media (Conrad et al., 2009; Charusanti et al., 2010; Wang et al., 2010; Maharjan et al., 2012; Saxer et al., 2014; Tenaillon et al., 2016), *E. coli* and *S. enterica* shared no overlap in mutation targets. For *E. coli*, the most common mutation target was in *rph-pyrE*, relieving a partial pyrimidine auxotrophy that is the largest limitation for this strain in M9^glu^ (Bonekamp et al., 1984; Jensen, 1993). *S. enterica* LT2 does not have this strain-specific defect, explaining why these mutants were only selected in *E. coli*.

*S. enterica*, on the other hand, acquired mutations in pyruvate kinase I (PykF). Growth with glucose as carbon source imparts a trade-off between using phosphoenolpyruvate (PEP) in glycolysis to generate ATP and pyruvate (catalyzed by pyruvate kinase) and for import of glucose through the PEP-dependent phosphotransferase system (PTS), which generates glucose-6-phosphate and pyruvate. During aerobic growth, the two ATPs per glucose that can be generated by PykF are negligible compared to the up to ~30 more ATPs generated through the other steps in glycolysis, the TCA cycle, and respiration, which probably favors efficient glucose import over maximum ATP yield from glycolysis.

### Complications of medium adaptation during fitness measurements

**Figure 8:**
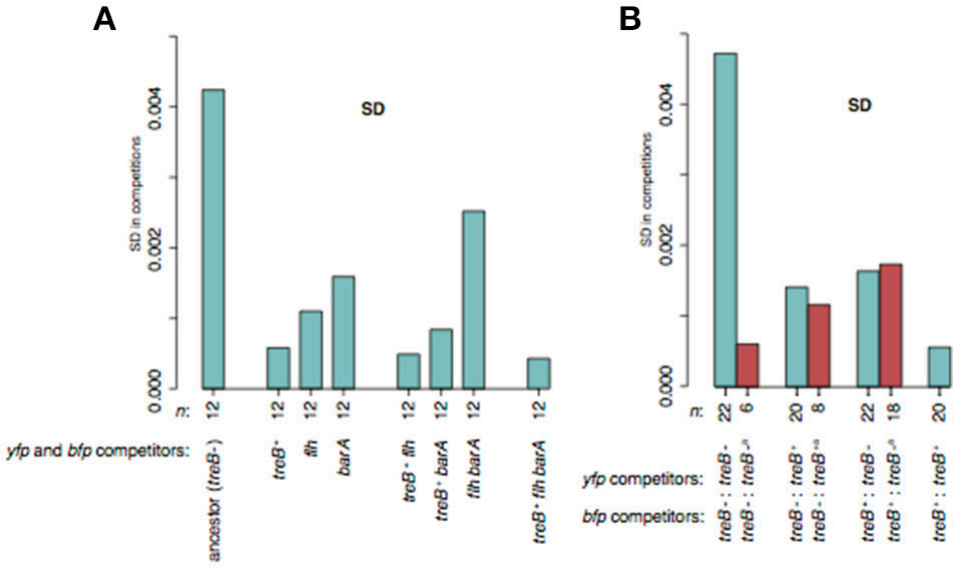
Variation in standard deviation (SD) of *S. enterica* LB mutants during competition experiments. **(A)** SD of competitions of *treB*^+^, Δ*flhE-D*, and Δ*barA* single and multiple mutants with the same mutant but with opposite marker. The reported values represent the SD between 12 independent biological replicates and no cultures were excluded. **(B)** SD in competitions of *treB*^−^ and *treB*^+^ mutants. Turquoise bars indicate the SD of 20 or 22 independent competitions whereas red bars indicate the SD when excluding competitions where spontaneous *treB*^+^ revertants were detected. The number of competitions for each SD measurement is indicated below the bars. See Figure S11 for fitness measurements.

We have frequently observed variation in competition experiments that we suspected were due to occurrence of spontaneous beneficial mutants in one of the competitors, resulting in a reduction in the accuracy of the fitness measurements. Based on the present study of media adaptation mutations, we considered the *treB*^+^-revertants, *barA* mutations, and flagellar mutations found in *S. enterica* evolved in LB as potential candidates for generating the fitness variation since they had large fitness effects and were detected in all evolved lineages (Tables S1, S2). To examine this idea, we performed competition experiments with twelve independent competitions of each *treB*^−^:*treB*^−^, *treB*^+^:*treB*^+^, Δ*flhE-D*:Δ*flhE-D*, and Δ*barA*:Δ*barA*, as well as the possible double and triple mutants (“*treB*^−^” is our un-evolved wild-type; Figure 8A and Figure S11). In line with our expectations, the standard deviation (SD) was lower for all mutants than for the *treB*^−^:*treB*^−^ competitions. The most dramatic decrease in SD was observed for mutants where *treB*^+^ was part of the genotype. In these cases, the SD of all three tested mutants was between 7- and 10-fold lower as compared to the *treB*^−^*:treB*^−^ competitions.

To further test if spontaneous *treB*^+^ mutants were the main cause of the variation in *S. enterica* competitions in LB, we competed *treB*^−^:*treB*^−^, *treB*^−^:*treB*^+^, and *treB*^+^:*treB*^+^ against the different competitors. In each pair, both configurations of the two fluorescent markers were run (Figure 8B and Figure S11). The standard deviations of *treB*^−^:*treB*^−^ and *treB*^+^:*treB*^+^ were similar to those measured in the previous experiment (4.73 × 10^−3^ vs. 4.24 × 10^−3^; and 5.6 × 10^−4^ vs. 5.8 × 10^−4^, respectively). To detect any spontaneous *treB*^+^ in the *treB*^−^:*treB*^−^ competitions, we plated the competitors on M9 trehalose plates on the final day of the competitions. On M9 trehalose, *treB*^+^ mutants would be detected as larger colonies (Figure S12). Spontaneous *treB*^+^ mutants were detected in some competing cultures, and after removing these from the calculations, the SD of the *treB*^−^:*treB*^−^ competitions was decreased from 4.73 × 10^−3^ to 6.7 × 10^−4^. This is similar to the 7-fold decrease in SD observed for *treB*^+^:*treB*^+^ competitions in comparison to *treB*^−^:*treB*^−^ competitions in the first experiment (Figure 8A). The cultures containing spontaneous *treB*^+^ mutants consistently showed a higher fitness than other cultures throughout the entire competition experiment and in all combinations where the same cultures were used. Likely, this reflects the selection for *treB* revertants in the ancestral colony or in the over-night culture, which was grown before mixing the competitors. In a similar manner, we also tested if a pre-existing *rph*^+^ mutation decreased SD in competitions with *E. coli* in MH (Figure S11). In this case, no large effects on SD were detected which might be because other media adaptation mutations contribute significantly to the variability.

In summary, these results show that media adaptation mutations can arise often enough to reduce the accuracy of fitness assays (Figure 8). Knowledge of which mutations appear in certain media, their frequencies and their impact on fitness can help to reduce the biological variation observed between replicate cultures/competitions. Thus, by discarding data from cultures that were shown to contain mutants with higher fitness, or by starting the experiment with an engineered parental strain that encodes the most common media adaptation mutation(s) with the largest effects, one may reduce the variation by 10-fold, allowing detection of fitness differences of |*s*| < 0.001. It is, however, important to consider effects such as epistasis between mutations before engineering parental strains for this purpose. It is possible that by further increasing the number of replicates (as was done in Gallet et al., 2012 study), even smaller fitness differences can be measured.

### Conclusions

We have identified a large set of medium adaptation mutations in *E. coli* and *S. enterica* and found that the spectrum of genes that are mutated in these two species and in different growth media are in a few cases similar but often differ substantially. These findings imply that for each species—medium combination, adaptation occurs via different genetic pathways and that even closely related species can show different evolutionary trajectories in response to identical growth conditions. The vast majority of the re-constituted and analyzed mutations were beneficial (on average an approximately 10% increase in fitness per mutation) under the conditions tested, and in most cases these effects were enhanced when multiple mutations were combined in the same strain. The mutant advantage in competition experiments and exponential growth rate generally correlated well for minimal media but less in complex media.

## Materials and methods

### Strains and media

*Escherichia coli* MG1655 (designated *E. coli* in the text) and *S. enterica* subsp. *enterica* serovar Typhimurium str. LT2 (designated *S. enterica*) were used as starting points for the evolution experiments and for re-constitution of the adaptive mutations. Deviations in our wild-type strains from the published genotypes (Blattner et al., 1997; McClelland et al., 2001) are described in Table S2. Phage P1 *vir* (Ikeda and Tomizawa, 1965) and P22 HT *int* (Schmieger, 1972) were used for transductions.

The liquid media used for the evolution experiment and for most fitness and relative exponential growth rate measurements were: LB (5 g yeast extract (Oxoid), 10 g Tryptone (Oxoid), 10 g NaCl L^−1^), MH (BD), and M9 minimal medium (Miller, 1992) supplemented with either 0.2% glucose, or 0.2% glycerol (designated M9^glu^ or M9^gly^). We have found that M9 medium prepared using Na_2_HPO_4_ other than Fluka's anhydrous Na_2_HPO_4_ (≥98%, pure, p.a.) results in medium that is deficient in iron (Table S6). For competitive fitness and relative exponential growth rate measurements of *malT* mutants we additionally used MH supplemented with 0.2% maltose or 4.5 g/l starch (approximately 4 × the normal amount in MH (BD). Similarly, for *treB* mutants LB supplemented with 0.2% trehalose was used, for *gltP* mutants LB supplemented with 0.5% glutamic acid, and for *sapD, sapF*, and *trkH* mutants with LB supplemented with 2.5 mM KCl. When preparing electro-competent cells, LB without salt was used. As solid medium, the LB, MH, M9^glu^, or M9^gly^ was supplemented with 15 g L^−1^ agar (LA). M9 supplemented with 15 g L^−1^ agar, and either 0.2% trehalose or 0.2% maltose was additionally used to detect *treB* and *mal* revertants. When needed, we used antibiotics in the concentrations 12.5 mg L^−1^ chloramphenicol, 50 or 100 mg L^−1^ ampicillin, and 7.5 mg L^−1^ tetracycline. To counter select against *sacB* cassettes, LA without NaCl, but supplemented with 50 g L^−1^ sucrose was used. All incubations were at 37 °C and with shaking if liquid media. For motility tests LB supplemented with 5 g L^−1^ agar was used (i.e., semi-solid medium). EBU plates (10 g L^−1^ Tryptone, 5 g L^−1^ Yeast Extract, 5 g L^−1^ NaCl, 2.5 g L^−1^ glucose, 15 g L^−1^ agar, 2.5 g L^−1^ K_2_HPO_4_, 0.00125% Evans Blue, and 0.0025% Sodium Fluoresceine) were used for cleaning *S. enterica* transductants from phage P22.

### Adaptive evolution

Independent cultures of *E. coli* and *S. enterica* were started from single colonies in four different media (LB, MH, M9^glu^, or M9^gly^; Table S2). In roughly 24 h intervals, the separate lineages were serially passaged by 1,000-fold dilution in 1 ml batch cultures, using 10 ml tubes (Sarstedt) except for *S. enterica* in M9^gly^, where the total volume was 1.5 ml. The carrying capacity in rich media is about 5–6 × 10^9^ cfu/ml, and slightly less in M9, about 1 × 10^9^ cfu/ml, resulting in bottlenecks of approximately 5 × 10^6^ cells per transfer in rich media, and 1 × 10^6^ cells per transfer for the minimal media. The effective population size is assumed to be *N*_*e*_ ≈ *gN*_0_, where *g* is the number of generations per transfer and *N*_0_ is the bottleneck (Lenski et al., 1991). Natural selection can act on |*s*| < 1/*N*_*e*_ (Kimura, 1983). Allowing for approximately 10 generations for each of our transfers, fitness advantages in the range of 10^−7^ could be seen by selection and the influence of drift is thus negligible. The number of days cycled for each of the 8 conditions (two species and four media) are found in Table S1. Some of these evolved lineages were already passaged and sequenced (as clones or populations) for other purposes and the populations were available in our strain collection (*S. enterica* LB, M9^gly^, and M9^glu^; and *E. coli* MH), while the remaining were serially passaged and sequenced (as populations) specifically for this study.

### WGS and sequence analyses

Between 4 and 12 evolved lineages from each condition (Table S1) were subjected to whole genome sequencing (WGS), using the facilities of BGI, Beijing, China (Illumina HiSeq), or our own Illumina MiSeq. As suggested by Dettman et al. (2012), most of the sequencing was done on the evolved populations and not on isolated clones (*E. coli* serially passaged in MH, *S. enterica* in LB, and two of the four *S. enterica* lineages evolved in MH were sequenced on isolated clones). The genomic DNA was prepared using Qiagen Genomic Tip 100/G or MasterPure^TM^ Epicentre. All bioinformatic analyses were done using CLC Genomics Workbench (CLC bio, Aarhus, Denmark). Illumina reads were trimmed using CLCs “Trim Reads” tool to remove any ambiguous positions, short reads (<15 nt), and low quality regions, using a stringency setting that trimmed the ends of most or all reads based on quality, after which the trimmed reads were mapped against a reference genome (for *S. enterica*, both the chromosome and the pSLT virulence plasmid were used as references. The average coverage of mapped reads over the chromosome varied between 61.4–74.5 × for the HiSeq sequencing (pSLT between 97–169 ×) and between 32–49 × (pSLT between 49–62 ×) for the MiSeq. To detect SNPs or indels, CLCs Low Frequency Variant Detection tool was used. Structural variations, such as transposition of insertion sequence (IS) elements, deletions, and inversions were found using the “Structural Variation” tool in CLC to find break points (partially mapped reads). The un-mapped ends of partially mapped reads were blasted against the reference genome to find where they matched. Transposition events were found as reads that were mapped to IS elements, but whose ends matched somewhere else in the reference genome, and as reads that were mapped to the site of the insertion but with un-mapped ends that matched the ends of an IS element in the reference genome. Fixed duplications or deletions were found by visual scanning of the mapped read depth, as regions with double or no coverage. We considered mutations with frequencies higher than 10% to be significant. The Illumina reads were deposited to the National Center for Biotechnology Information Sequence Read Archive with BioProject ID PRJNA433618.

### Re-constitution of evolved mutants

Firstly, the desired mutations were screened for by isolating separate clones from the evolved populations, followed by PCR and sequencing over the position for the mutation. In order to re-constitute the mutations (in a clean background and with no marker or “scar sequence”) we used Dup-In recombineering (Näsvall et al., 2017) for the majority of the mutations. Shortly, by duplicating an area (400–1,000 bp) in near proximity to (or including) the mutations (co-transducible), and at the same time inserting a counter selectable marker, the duplications and the mutations could easily be moved through transduction to the desired background. Once in the new background, the duplication was segregated and the mutation (“scar-free”) was left.

For other mutations, we instead used a “two-step” protocol, where we first inserted a *cat-sacB* (GenBank KM018298) or an *amilCP*-*cat*-*sacB* (*Acatsac1*; MF124798, Näsvall et al., 2017) cassette linked to the mutations by λ red recombineering (Ellis et al., 2001). The mutations and the counter selectable cassettes were then moved to the desired background by transduction. Thereafter, the counter selectable cassette was removed by back-transduction from the strain carrying the desired mutation but not the *cat-sacB* or *Acatsac1* cassettes, followed by selection on sucrose. The resultant strain was, in similarity to the final strain after Dup-in recombineering both marker free and “scar-free.”

In the case of the *rph-pyrE* locus, we chose to generate a true reversion of the *rph-1* mutation instead of using one of the evolved alleles. To do this, we used the DIRex method (Näsvall, 2017). Briefly, a *cat-sacB* cassette containing a large inverted repeat (<*AcatsacA*>) was inserted between two copies of identical short directly repeated sequences, containing the corrected sequence. The presence of the large inverted repeat stimulated loss of the *cat-sacB* cassette, leaving the corrected sequence in *rph*. The same method was used to create the *rpoS*(Ala130Glu), *clpA*(Thr354Met) and *malQ*(Arg96Leu) mutations.

The deletion mutants were performed by first exchanging the gene with a *cat-sacB* cassette through λ red recombineering (Ellis et al., 2001). Then the *cat-sacB* in turn was exchanged by an oligo containing 35 bp upstream of the gene plus the first 2–3 codons, followed by the last 2–3 codons and 35 bp downstream of the gene. This creates an in-frame deletion. Next, the deletion was moved using the Dup-In method described above.

All the final strains have been sequenced for the desired mutations and in the cases where a *cat-sacB* or *Acatsac1* cassette was exchanged by an oligo, loss of the cassettes was determined by PCR. All primers used in the constructions are found in Tables S7, S8 and complete strain lists are found in Tables S2, S4. Table S4 also specifies which method was used for re-construction of each single mutant. The Dup-In and DIRex methods (Näsvall, 2017; Näsvall et al., 2017) were developed during the period of this project, resulting in a transition between different methods.

Mutations were transferred between strains using generalized transduction with phage P1 (*E. coli*) and P22 (*S. enterica*). P1 lysates were prepared, and transductions were performed as described (Thomason et al., 2007). P22 transducing lysates were prepared by mixing 200 μl overnight culture of the donor strain with 1 × 10^6^ pfu P22 (grown on the wild-type strain) in 1 ml LB. When lysis was evident, any remaining cells were killed by adding chloroform and cell debris was pelleted through centrifugation. 0.5 μl transducing lysate was mixed with 200 μl overnight culture of the recipient strain and was incubated at 37°C for 30 min before plating on selective media. Transductants were purified from phages by streaking on EBU plates, which contains pH indicators that help distinguishing uninfected colonies from infected colonies.

### Construction of strains for competitions

Chromosomal copies of the fluorescent protein genes *bfp* (*mtagbfp2*, blue; Subach et al., 2011; Gullberg et al., 2014) and *yfp* (*syfp2*, yellow; Kremers et al., 2006; Gullberg et al., 2014) were moved with P1 or P22 transduction into the re-constituted strains. The two fluorescent protein genes were inserted in the *galK*-locus of both *E. coli* and *Salmonella*.

### Competition experiments

Competition experiments to measure relative fitness were performed by tagging the mutant and an isogenic wild-type control strain (the ancestral parent) with two different fluorescent markers and mixing those 1:1. The two were then serially passaged together for 4 days under the same conditions as under the evolution experiment (except for *S. enterica* in M9^gly^, where 1 ml culture volumes were used instead of 1.5 ml). At days two, three, and four the ratio of mutant to wild-type was measured by counting 10^5^ fluorescent cells, using a flow cytometer. Six independent competitions were performed for each mutant, where three were the competition between a wild-type carrying the *yfp* marker and the mutant carrying the *bfp* marker, and three with the opposite marker. Clear outliers were omitted. In most experiments there was a slight fitness difference between the *yfp* and *bfp* markers, but the size and direction varied between experiments, conditions and strains. Hence we did not correct for this cost. Selection coefficients (*s*) were determined using the regression model, *s* = ln(R[t]/R[0])/t (Dykhuizen, 1990), where R is the ratio of mutant to wild-type and t is number of generations.

### Effect of adaptive mutations on variation

To study the negative effect of spontaneous adaptive mutations on fitness measurements we conducted a competition experiment where eleven *yfp*-marked independent *S. enterica* wild-type cultures that originated from single colonies were competed against two independent wild-type cultures marked with *bfp* (Figure 8B). We additionally competed the two wild-type *bfp* cultures with eleven *treB*^+^ *yfp* and also competed the same eleven *treB*^+^ *yfp* cultures with two independent *treB*^+^ *bfp* cultures (total 22 competitions of each of the three types). We competed the cultures for 4 days (approximately 40 generations). Apart from measuring *s*, 100 μl of a 6 × 10^5^-fold dilution of the competing cultures were plated on M9 trehalose plates in order to estimate the frequency of *treB*^+^ revertants in the wild-type to wild-type competitions. TreB^+^ revertants were detected as larger colonies among ~700–1,300 tiny colonies. For two out of the 22 *treB*^+^ to *treB*^+^ competitions (same *yfp* competitor, different *bfp*), the *s*-value clearly deviated from the remaining 20 competitions and fell well outside the interquartile range wherefore we decided to remove these. When removed, the variation was very close to the corrected wt:wt variation (0.00056 and 0.00067, respectively) and was also very similar to the *treB*^+^ to *treB*^+^ competitions performed for Figure 8A. We do not know why this clone of the *yfp* competitor had reduced fitness.

A similar experiment was done for twelve Δ*flhE-D yfp* against twelve Δ*flhE-D bfp*, and so on, including the single mutants *treB*^+^, Δ*flhE-D*, Δ*barA*, as well as the possible double and triple mutants (Figure 8A). For this experiment, only competitive fitness was measured.

### Relative exponential growth rate measurements

Relative maximum exponential growth rate measurements were performed as described previously (Knöppel et al., 2016). Briefly, the optical density at OD_600_ was measured every 4 min, using a Bioscreen C Reader (Oy Growth Curves) or an Infinite 200 PRO (Tecan). The maximum exponential growth rates were calculated and normalized to the maximum exponential growth of isogenic wild-type controls included in each experiment. Reported values correspond to the average of between two and eight (in most cases four) independent biological replicates ± standard deviation.

### Statistical analysis

Pearson correlation coefficients were calculated using the online tool at http://www.socscistatistics.com/pvalues/pearsondistribution.aspx.

To test for epistatic interactions between two alleles we assumed a null hypothesis of no epistasis (*i*.*e*., purely additive effects). The expected fitness for the double mutants were calculated from the observed fitness of the single mutants:

$$\begin{array}{l} s_{ab}^{\exp}=s_{a}^{obs}\times s_{b}^{obs} \end{array}$$

where *s* is relative fitness, and *a* and *b* denote the mutant alleles, and the standard deviations (σ) were propagated:

$$\sigma_{ab}^{\exp}\;=\;\sqrt{{\left(s_{a}^{obs}\times \sigma_{b}^{obs}\right)}^{2}+{\left(s_{b}^{obs}\times \sigma_{a}^{obs}\right)}^{2}}$$

If the expected fitness (with standard deviations) fell within two standard deviations of the observed fitness, the effects could not be distinguished from simple additivity. Thus, positive epistasis was called if $s_{ab}^{obs}-2\times \sigma_{ab}^{obs}>s_{ab}^{\exp}+\sigma_{ab}^{\exp}\;$and negative epistasis if $s_{ab}^{obs}+2\times \sigma_{ab}^{obs}<s_{ab}^{\exp}-\sigma_{ab}^{\exp}$.

## Author contributions

AK, JN, and DA designed and directed the study. AK performed most experiments. MK, LA, EL, and UL performed strain cycling and JN analyzed whole genome sequences and performed the experiments for Figure 8, Figures S7, S8, S12. AK designed the figures. AK, JN, and DA wrote the manuscript.

### Conflict of interest statement

The authors declare that the research was conducted in the absence of any commercial or financial relationships that could be construed as a potential conflict of interest.
