# Supplementary material for: Genetic Adaptation to Growth Under Laboratory Conditions in Escherichia coli and Salmonella enterica
Source: Front Microbiol. 2018 Apr 26;9:756. doi: 10.3389/fmicb.2018.00756 (PMC5933015; doi:10.3389/fmicb.2018.00756)
Supplement: Supplementary file 1 [file Image_1.pdf]

Supplementary Material

Genetic adaptations to growth under laboratory conditions in *Escherichia coli* and *Salmonella enterica*

Anna Knöppel, Michael Knopp, Lisa Albrecht, Erik Lundin, Ulrika Lustig, Joakim Näsvall, and Dan I. Andersson*

*** Correspondence:** Dan I. Andersson: dan.andersson@imbim.uu.se

Figures S1 – S12

Supplementary Discussion

Supplementary References

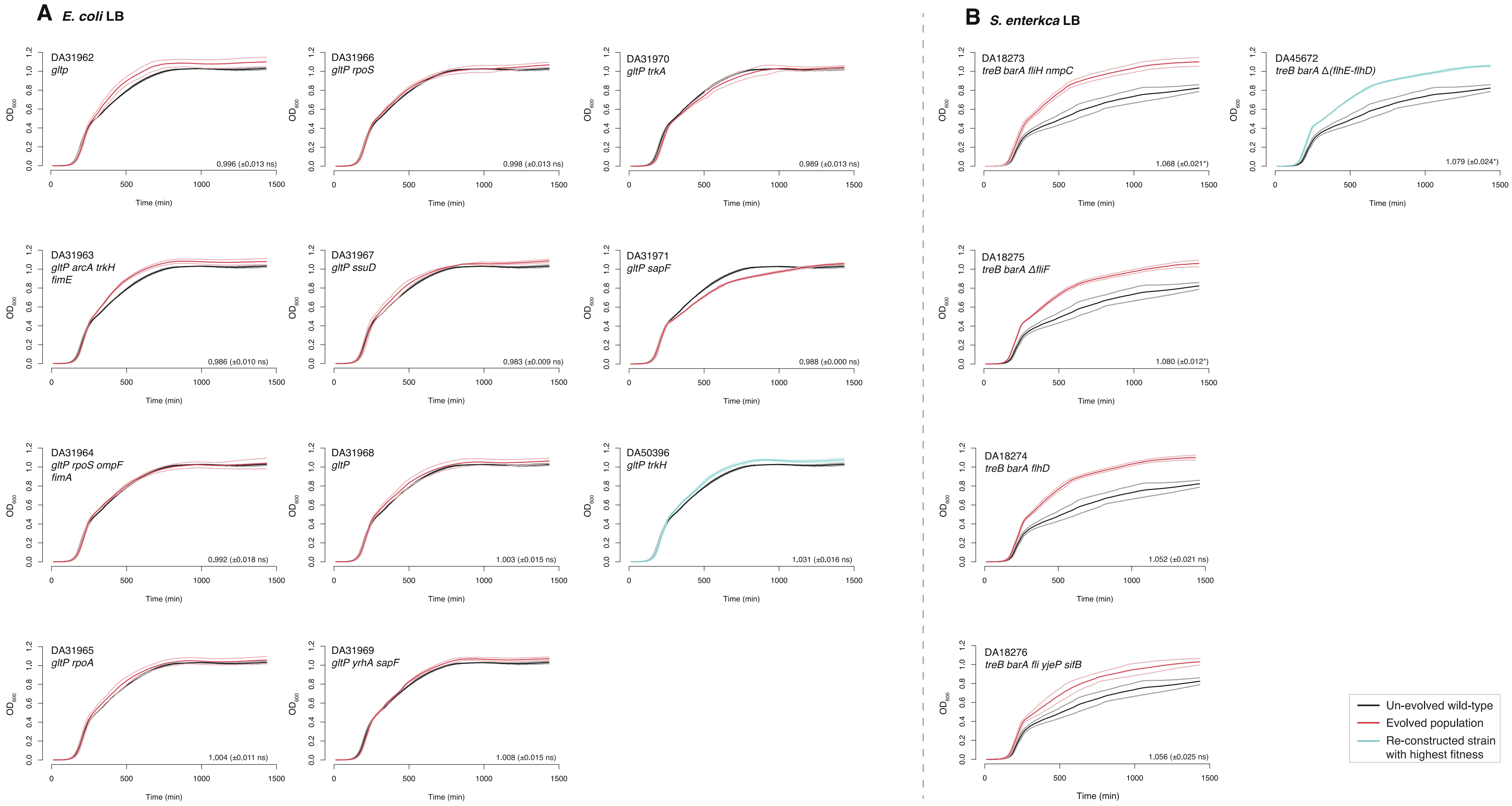

FIG. S1. Growth curves for lineages evolved in LB (red). The growth of the un-evolved wild-type control strains are included for comparison (black). The last graph in each panel shows the growth of the re-constructed strain with the highest measured competitive fitness (turquoise). In order to adjust for the effects of different starting ODs on the apparent lag time, the time point where the culture OD reached 0.006 was set to T = 0 min. Note that this removes any real differences in lag time. The thick lines are the averages of 2 – 3 replicates, and the thinner lines are the standard deviations. Genes found to be mutated are indicated for each population (see Supplementary Table S2 for exact mutations).

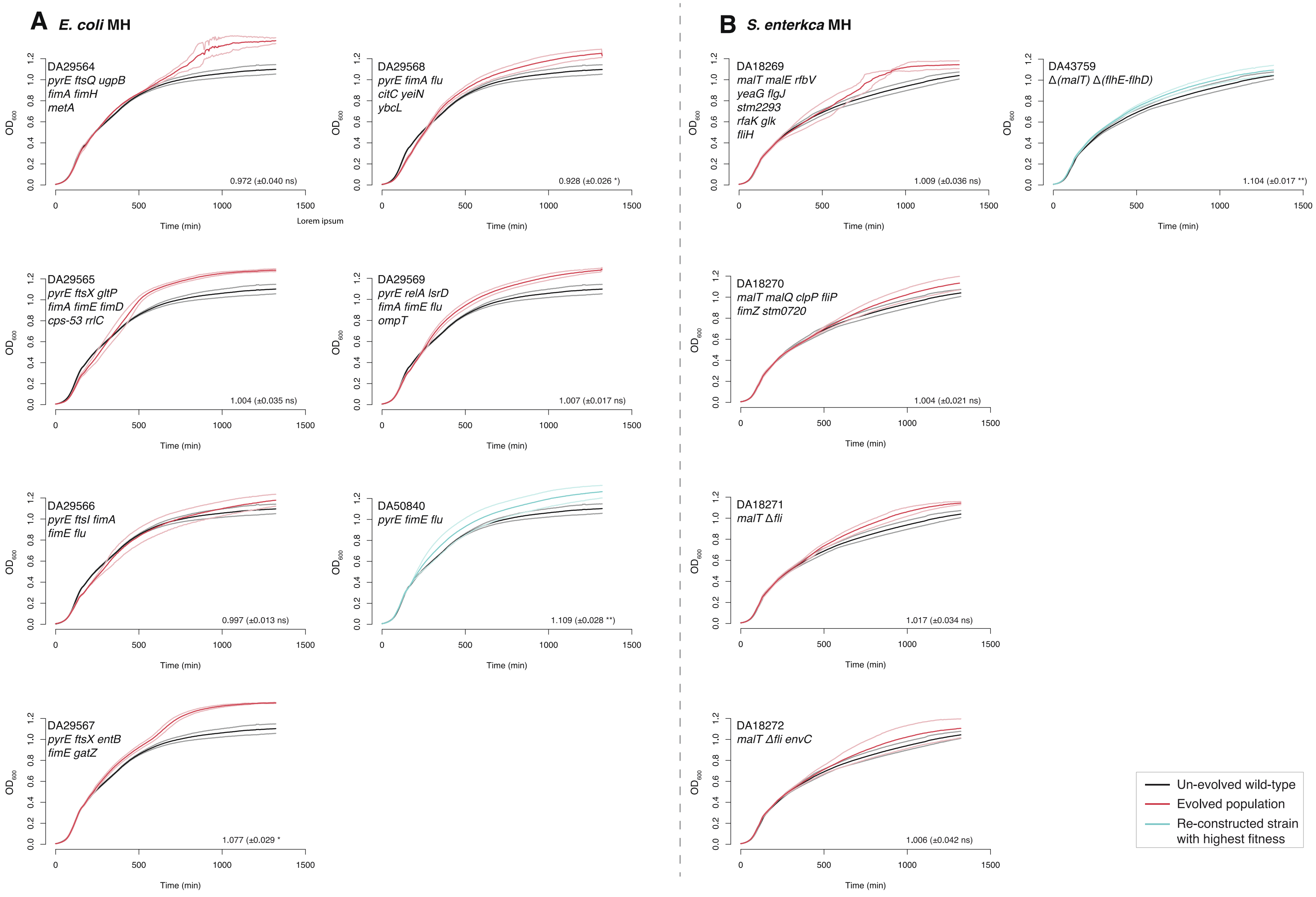

FIG. S2. Growth curves for lineages evolved in MH (red). The growth of the un-evolved wild-type control strains are included for comparison (black). The last graph in each panel shows the growth of the re-constructed strain with the highest measured competitive fitness (turquoise). In order to adjust for the effects of different starting ODs on the apparent lag time, the time point where the culture OD reached 0.006 was set to T = 0 min. The thick lines are the averages of 2 – 3 replicates, and the thinner lines are the standard deviations. Genes found to be mutated are indicated for each population (see Supplementary Table S2 for exact mutations).

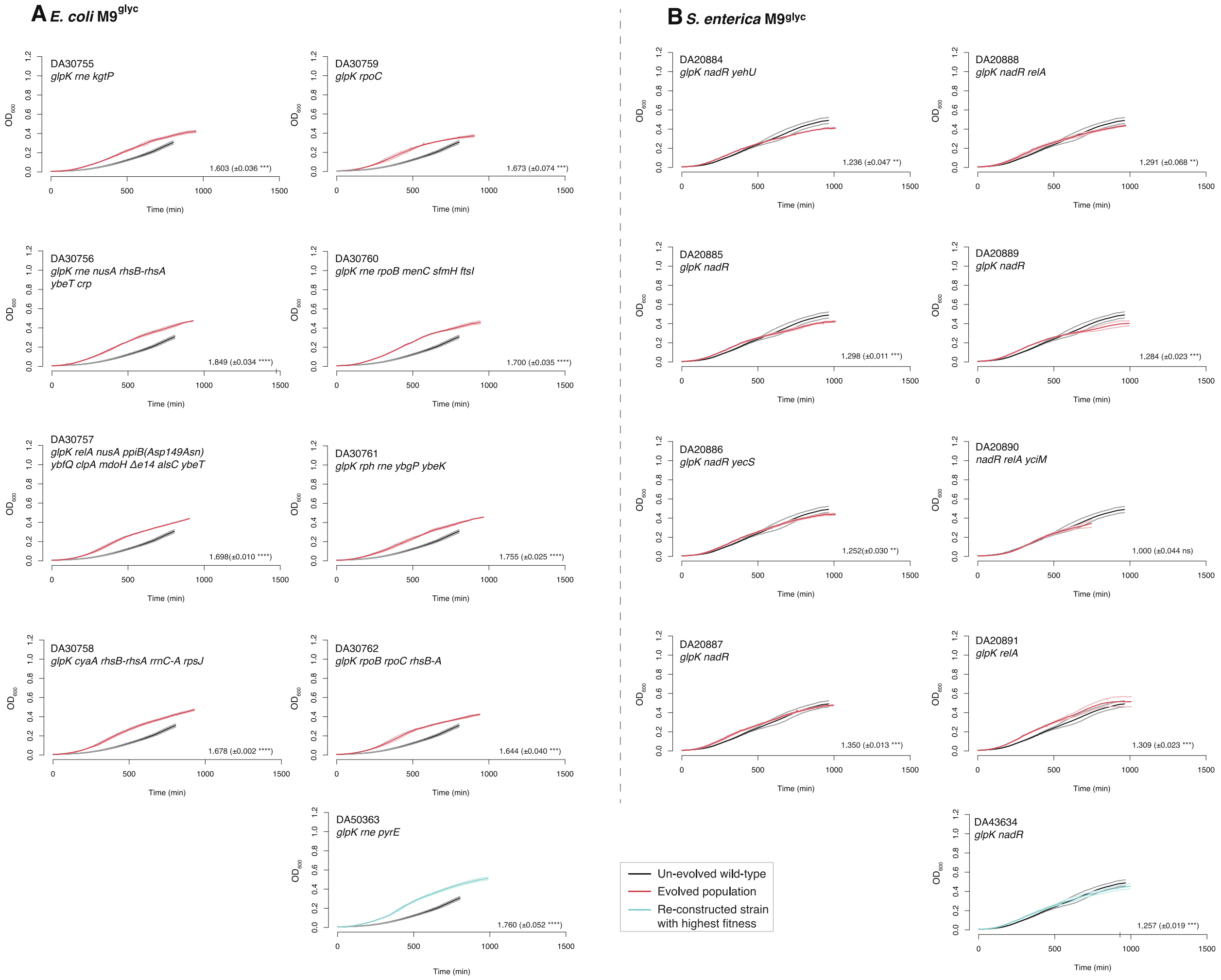

FIG. S3. Growth curves for lineages evolved in M9 glycerol (red). The growth of the un-evolved wild-type control strains are included for comparison (black). The last graph in each panel shows the growth of the re-constructed strain with the highest measured competitive fitness (turquoise). In order to adjust for the effects of different starting ODs on the apparent lag time, the time point where the culture OD reached 0.006 was set to T = 0 min. The thick lines are the averages of 2 – 3 replicates, and the thinner lines are the standard deviations. Genes found to be mutated are indicated for each population (see Supplementary Table S2 for exact mutations).

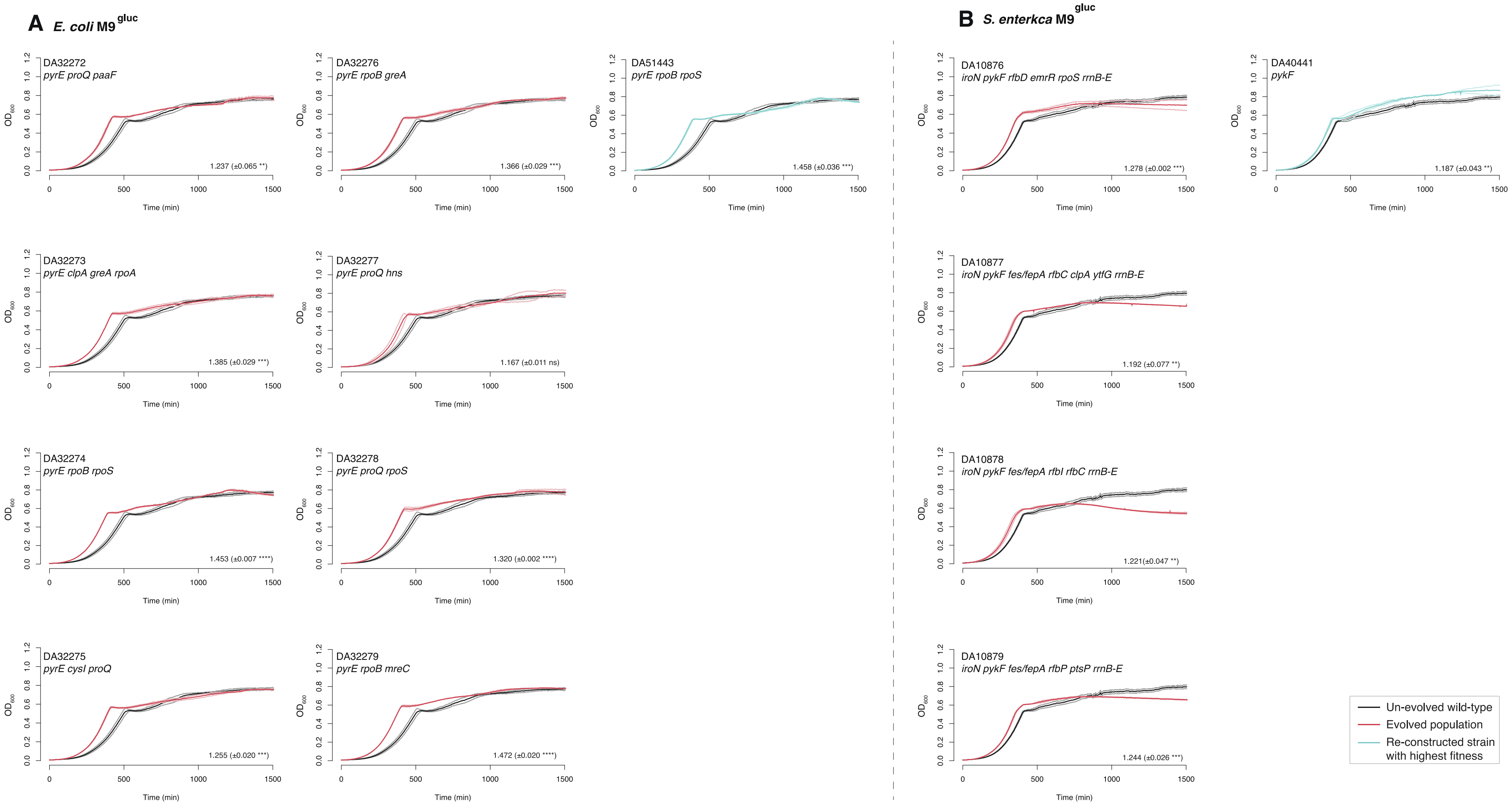

FIG. S4. Growth curves for lineages evolved in M9 glucose (red). The growth of the un-evolved wild-type control strains are included for comparison (black). The last graph in each panel shows the growth of the re-constructed strain with the highest measured competitive fitness (turquoise). In order to adjust for the effects of different starting ODs on the apparent lag time, the time point where the culture OD reached 0.006 was set to T = 0 min. The thick lines are the averages of 2 – 3 replicates, and the thinner lines are the standard deviations. Genes found to be mutated are indicated for each population (see Supplementary Table S2 for exact mutations).

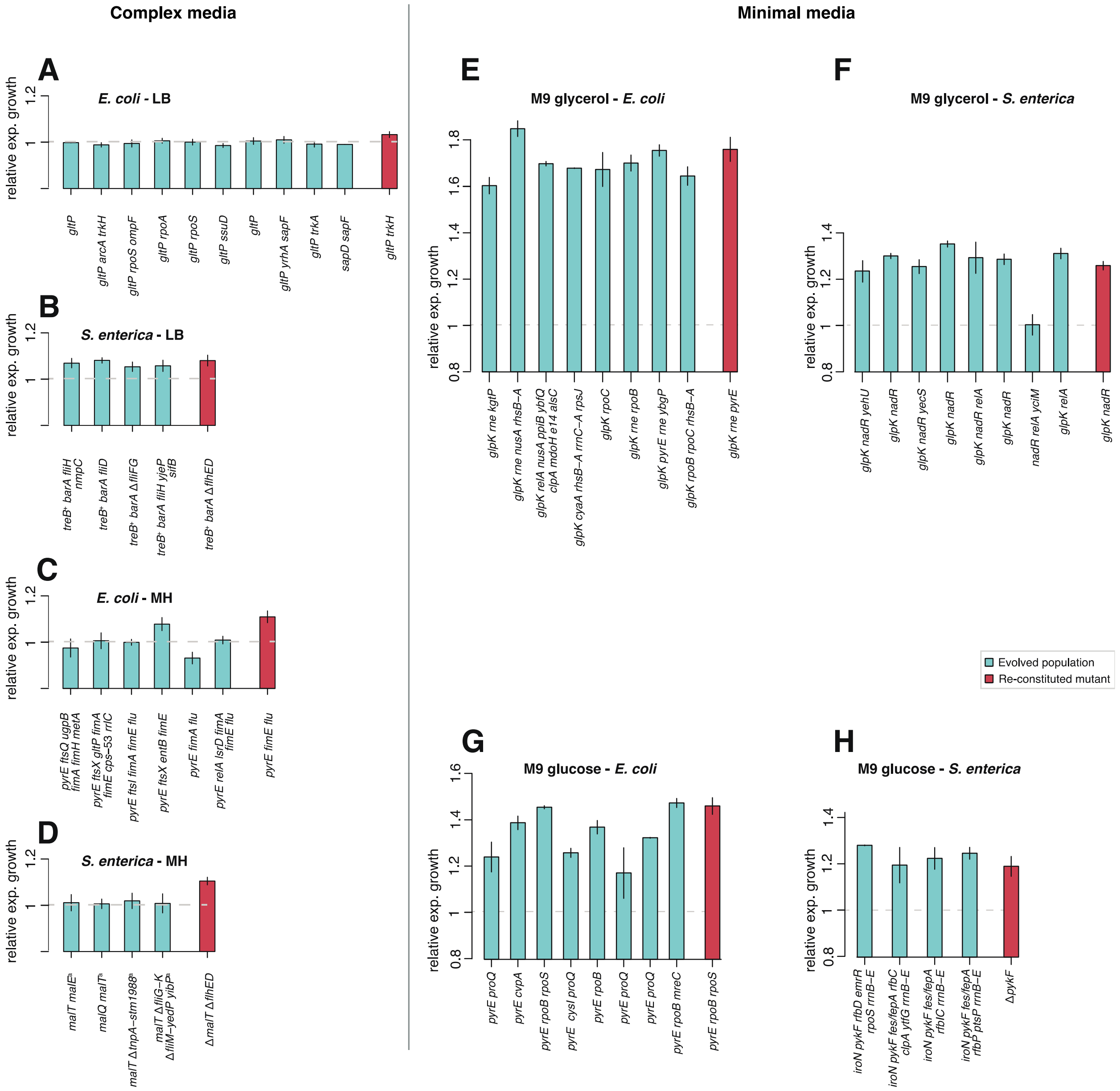

FIG. S5. Relative exponential growth rates (±SD) measured for evolved populations. (A) *E. coli* LB, (B) *S. enterica* LB, (C) *E. coli* MH, (D) *S. enterica* MH, (E) *E. coli* M9 glycerol, (F) *S. enterica* M9 glycerol, (G) *E. coli* M9 glucose, and (H) *S. enterica* M9 glucose. The re-constituted multiple mutant with the highest measured exponential growth rate was included for comparison (red bars). The evolved populations are arranged in the same order as in Tables S1 and S2. Growth curves are found in Supplementary Fig. S1 – S4.

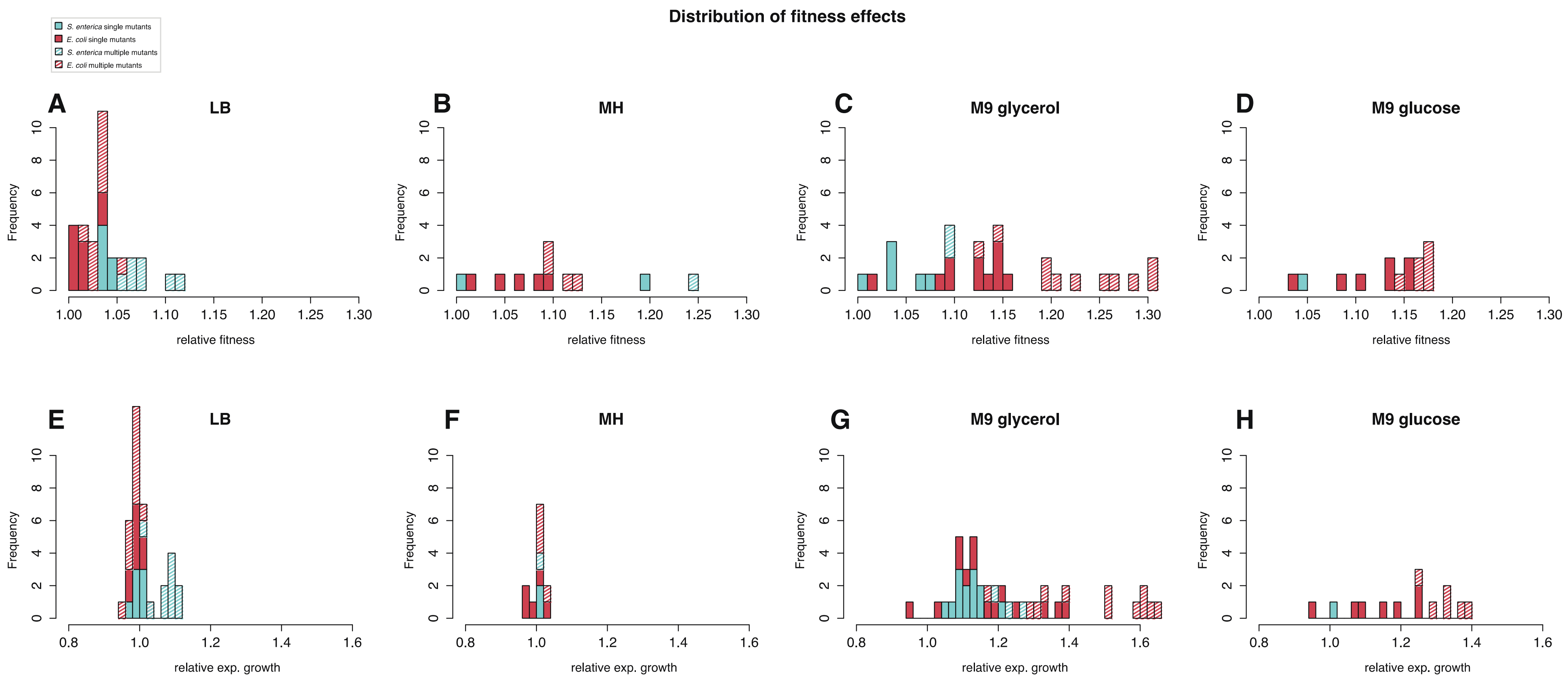

FIG. S6. (A – D) Distribution of fitness effects in the different media. Turquoise bars, *S. enterica* single mutations; red bars, *E. coli* single mutations, turquoise striped bars *S. enterica* multiple mutations; red striped bars *E. coli* multiple mutations. (E – H) As in A – D but relative exponential growth rate.

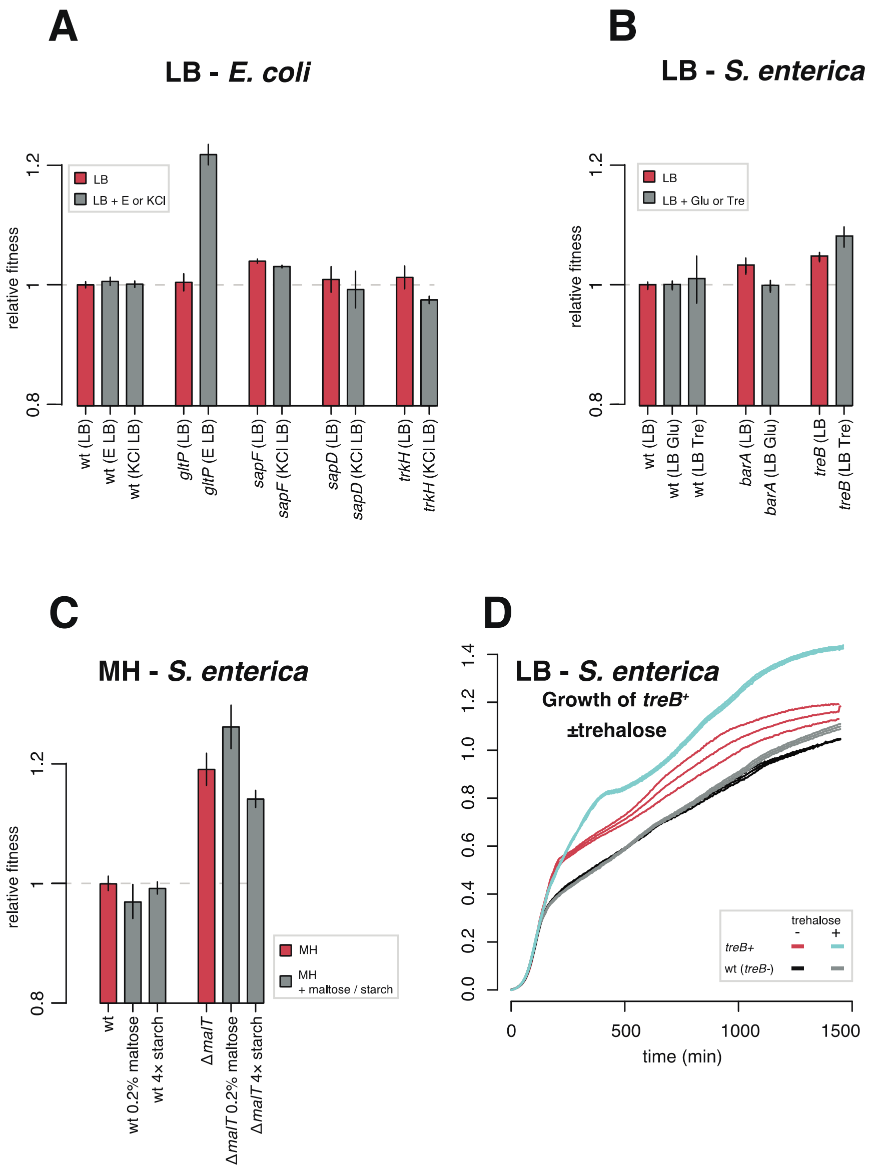

FIG. S7. Relative competitive fitness measurements ±SD with and without supplements to the growth medium (gray = with; red = without). (A) LB *E. coli*. The *gltP*(C-118del) mutant (w/v) 0.5% glutamate, and the *sapF*(Arg158Ser), *sapD*(Gly235Ser), and *trkH*(Pro94Ser) mutants (w/v) 2.5 mM KCl. (B) LB *S. enterica*. The Δ*barA* and *treB*$^{+}$ mutants (w/v) 0.2% glucose or 0.2% trehalose, respectively. (C) MH *S. enterica*. The Δ*malT* mutant (w/v) 0.2% maltose or 4× starch (final conc. 6 g/L). (D) Growth curve for the *treB*$^{+}$ mutant (red) and wild-type (*treB*$^{-}$; black) grown in LB, and the same strains grown in LB supplemented with 0.2% trehalose (turquoise and grey, respectively. In order to adjust for the effects of different starting ODs on the apparent lag time, the time point where the culture OD reached 0.006 was set to T = 0 min. The lines represent the averages and standard deviations of 2 – 3 replicates.

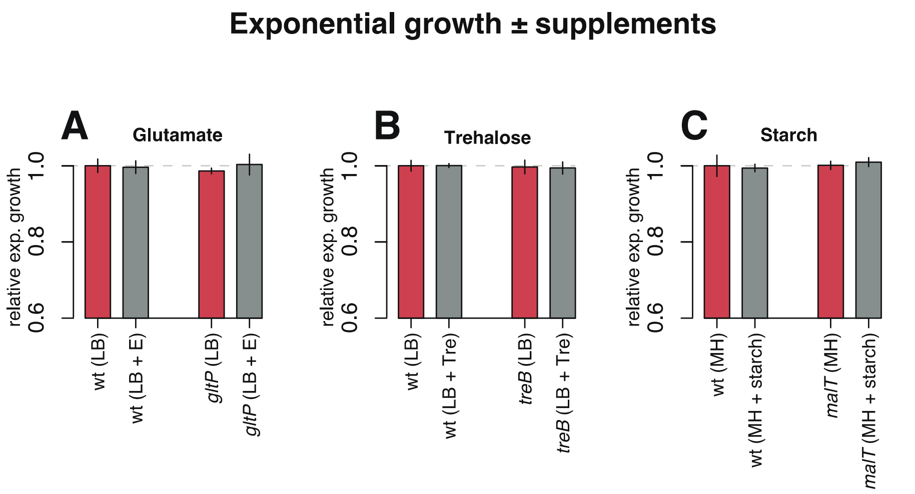

FIG. S8. Relative maximum exponential growth rates (±SD) of wild-type and mutant strains with and without different supplements. (A) *E. coli* wild-type and *gltP*(C-118del) in LB ± 40 mM glutamate (indicated with "E"). (B) *S. enterica* wild-type and *treB*$^{+}$ grown in LB ± 0.2 % trehalose. (C) As in A but *S. enterica* Δ*malT* MH ± 4× starch (4.5g/L).

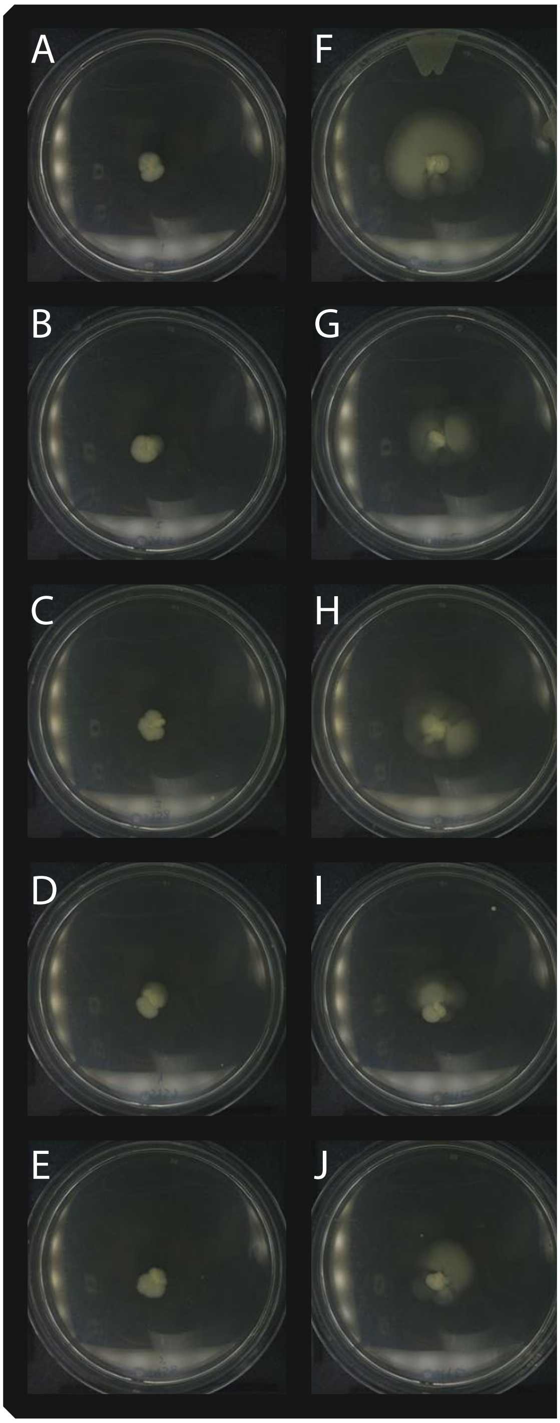

FIG. S9. The ancestral *S. enterica* strain (DA6192) is more motile than the ancestral *E. coli* strain (DA5438) in LB medium. Fresh single colonies were picked from LA plates using a plastic inoculation loop, and used to stab bacteria in the center of a soft agar LB plate (LB with 0.33% agar). (A - E) DA5438, *E. coli*. (F - J) DA6192, *S. enterica*. The plates were photographed after approximately 25 hours at 37°C.

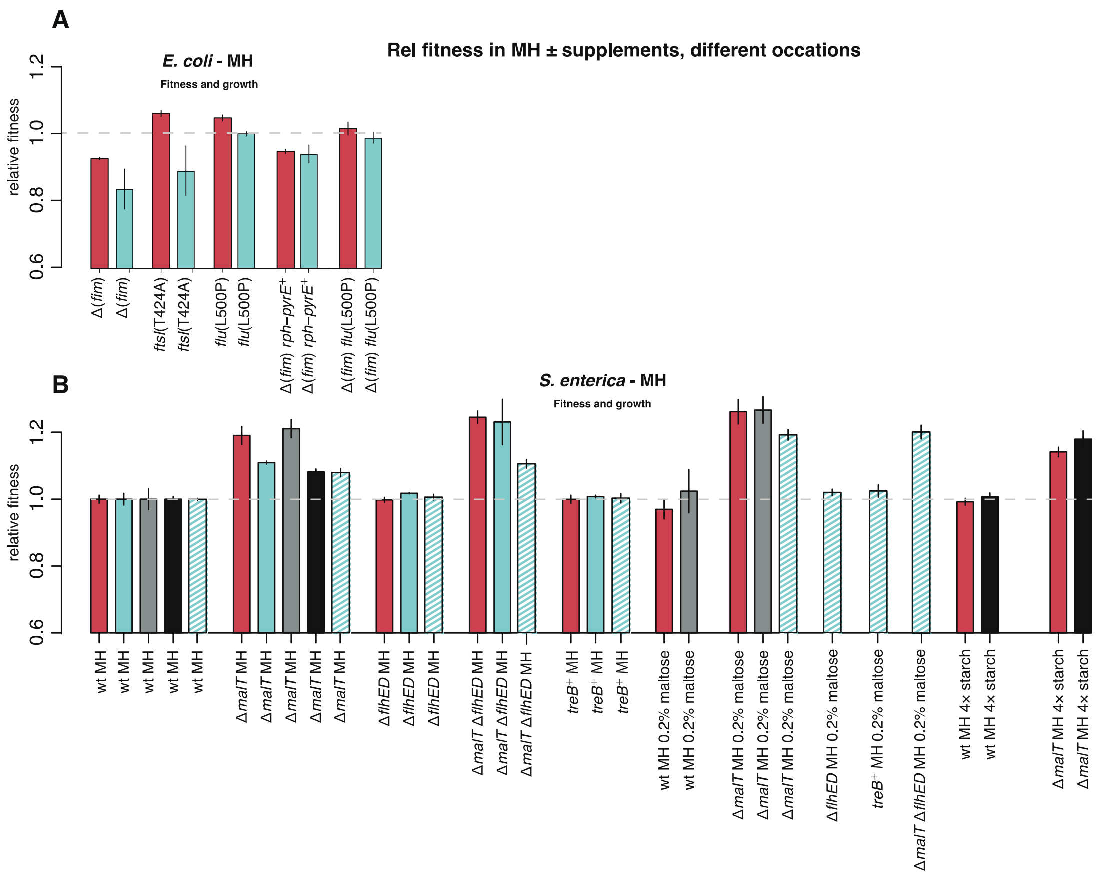

FIG. S10. Relative competitive fitness of re-constituted mutants, measured at different occasions. (A) *E. coli* mutants measured at different occasions with two different medium batches per strain. (B) *S. enterica* mutants measured at five different occasions with different media batches each time. Here the five different colors (red, turquoise, grey, black, and turquoise stripes) represent five different time points of measurements and five different batches of MH medium for *E. coli* (A) an *S. enterica* (B), respectively. The red bars represent the same experiment as shown in Fig. 5B and D in the main text.

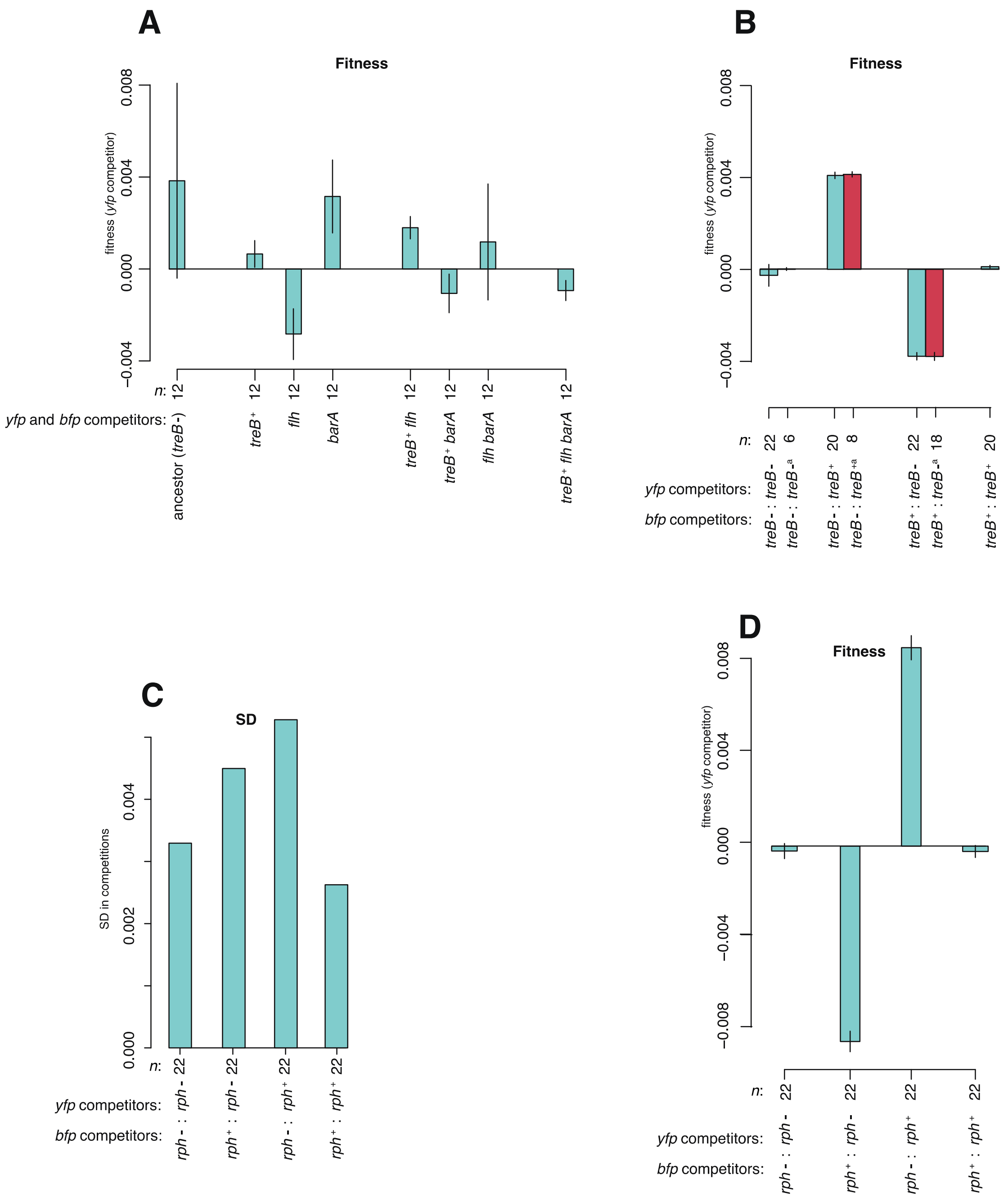

FIG. S11. (A) Relative competitive fitness (±SD of 12 replicate competitions) of the same experiment as in Fig. 8A. (B) Relative competitive fitness (±SD) of the same experiment as in Fig. 8B. Turquoise bars indicate the SD of 20 or 22 independent competitions whereas red bars indicate the SD when excluding competitions where spontaneous *treB*$^{+}$ revertants were detected. The number of competitions for each SD measurement is indicated below the bars. (C) Variations in standard deviation (SD) of *E. coli* MH *rph*$^{+}$ mutants during competition experiments. The number of competitions for each measurement is indicated below the bars. (D) Relative competitive fitness (±SD) of the same experiment as in C.

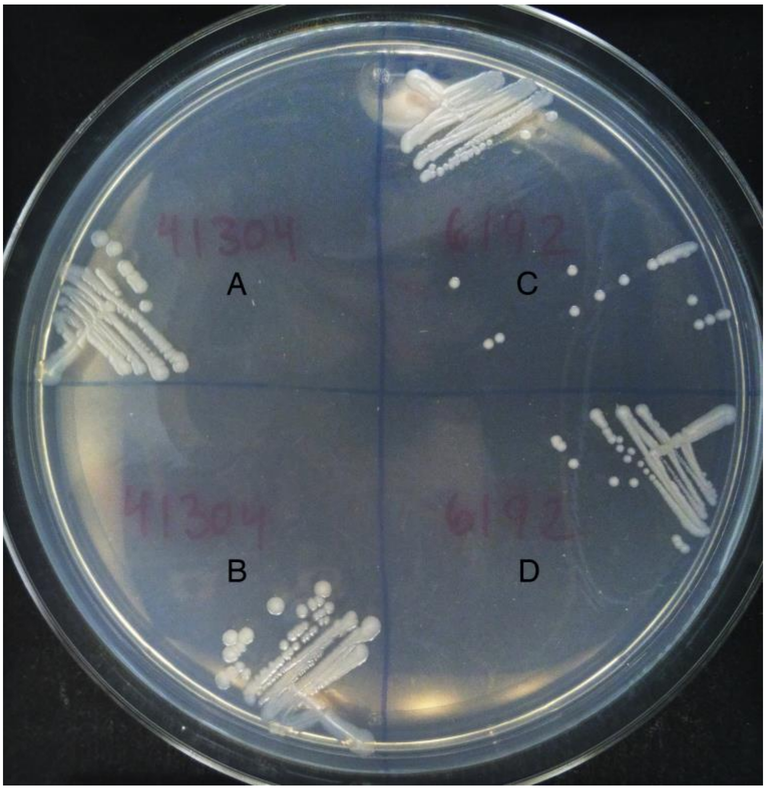

FIG. S12. Growth of *S. enterica* on M9 + 0.2% trehalose. (A and B) A re-constructed *treB*$^+$ revertant (DA41304). (C and D) The *treB*$^-$ ancestor (DA6192). The plate was incubated for two days at 37 °C.

Supplementary Discussion

Evolution to the four media

LB

Adaptation to LB in *E. coli* was characterized by few mutations in the evolved lineages (on average 2.6) and low increases of fitness of the re-constructed mutants (Fig. 5A). On the other hand, *S. enterica* had slightly more mutations (on average 3.8) per evolved lineage and higher relative fitness of the re-constructed mutants (*t*-test: $p < 10^{-4}$). In accordance, the relative exponential growth rates of the evolved lineages were lower for *E. coli* than for *S. enterica* (on average 0.99 and 1.06, respectively; *t*-test: $p < 10^{-5}$; Supplementary Fig. S5). No overlap between the mutational targets for the two species was found and it is plausible that the *E. coli* lab strain used, MG1655, had already gone through substantial amounts of adaptation to growth in LB since its isolation in 1922 (Hayashi et al., 2006). In support of this, Saxer et al. (2014) discovered 26-fold more mutations than we did in WGS parallel lineages of a naïve isolate of *E. coli* (disregarding mutators) that had been serially passaged in LB for approximately 500 generations (competitive fitness was not assessed in that study).

MH

For MH, the relative exponential growth rates of the evolved populations similar to the un-evolved wild-types for both *E. coli* and *S. enterica* (1.00 and 1.01, respectively; *t*-test: $p > 0.05$) and the mutation targets differed between *E. coli* and *S. enterica* that were serially passaged in MH (Table S1; Fig. 5C and D). However, in comparison to LB, more mutations were found per lineage (on average 6.6 and 5.3 in *E. coli* and *S. enterica*, respectively) and the fitness increases in the re-constructed mutants were higher. In *E. coli*, strong parallelism between the six lineages was seen for mutations that restore expression of *pyrE*, thus relieving the poor pyrimidine synthesis in *E. coli* K12 (further discussed below) as well as various mutations affecting one or several fimbrial genes. In addition, different genes in the cell division machinery were mutated in four lineages, and mutations in the phase variable biofilm formation gene *flu* (*agn43*) and its promoter (Wallecha et al., 2014) were found in three strains. The highest increase in competitive fitness among the single mutants in *E. coli* was detected for a constructed *rph* frameshift reversion ($s = 1.09$) and only little or no increase was seen when introducing additional mutations into the *rph-pyrE*$^{+}$ strain (Fig. 5C).

M9^{gly}

E. coli and *S. enterica* evolved in M9 glycerol displayed the largest increase in relative exponential growth rate out of all four media, although *E. coli* showed a much stronger increase than *S. enterica* (on average 1.70 and 1.25, respectively; Fig. 6A – B). The most common mutation for both species was amino acid substitutions in the *glpK* gene, encoding a key enzyme for glycerol metabolism. The two species even shared mutations in amino acid position Val8 and Arg34. GlpK catalyzes the first step in glycerol catabolism and is a known target for mutations in *E. coli* B and K12 evolved in M9 glycerol (Herring et al. 2006; Applebee et al. 2011; Le Gac et al. 2013).

In both species, some re-constructed *relA* mutations conferred faster exponential growth rates (excluding Δ*relA*), but a detectable increase in competitive fitness could only be found in *E. coli* (Fig. 6A and B). Thus, for *S. enterica* the correlation between relative fitness and relative exponential growth rate was weak when all mutants were included but strong when excluding *relA* (Fig. 4B). It is unclear why we could not detect any benefit of the *relA* mutations tested in *S. enterica*, but it is possible that they were beneficial only in competition with mutants that were present in the same populations at some point during the evolution experiment. *relA* mutations have previously been found in *E. coli* B adapted to glucose minimal medium (Le Gac et al. 2013).

M9^{glu}

E. coli and *S. enterica* evolved in M9 glucose showed similar increases in relative exponential growth (on average 1.33 and 1.23, respectively; *t*-test: $p > 0.05$; Fig. 6C and D). One mutational target (*rpoS,* encoding the stress response RNA Sigma factor S), overlapped between the two species and was previously described for multiple species evolved in complex and minimal media (Charusanti et al., 2010; Conrad et al., 2009; Maharjan et al., 2012; Saxer et al., 2014; Tenaillon et al., 2016; Wang et al., 2010).

In *S. enterica,* all four lineages had acquired mutations that could be associated with iron deficiency (*e.g. iroN* and *fes*/*fepA*; Zorraquino et al. 2017). Since iron is not added specifically during preparation of M9 medium, it has to be present as a contaminant in some component. We have found that some sources of Na_2HPO_4 results in medium that does not allow growth to full density unless Fe^{2+} is added (See Materials and Methods and Supplementary Table S6). These lineages were evolved before this observation was made, so it was very likely that the iron metabolism mutations were selected in an iron deficient batch of M9, which is why we decided to characterize only the *clpA*(Thr354Met) mutation and a constructed Δ*pykF* mutant.

Possible mechanistic causes of the beneficial effects

LB – *E. coli*

gltP

The most frequently mutated gene in *E. coli* grown in LB (found in 9 out of 10 populations) was the glutamate/aspartate proton symporter *gltP.* Two different *gltP* promoter mutations were found (A-115T and -118ΔC). Surprisingly for such a repeatedly occurring mutation, the re-constituted -118ΔC mutation showed no detectable fitness increase in competition experiments, except for a small increase in combination with other mutations (Fig. 5A) and, although unlikely, we cannot completely rule out the possibility that this mutation was already present in the frozen stock culture of the wild-type. However, when L-glutamate was added to the medium to a final concentration of 40 mM, the mutants revealed a strong increase in relative fitness as compared to no added L-glutamate ($s^{+glutamate} = 1.22$, $s^{-glutamate} = 1.00$; Supplementary Fig. S7A). No effect of L-glutamate was detected for neither wild-type nor mutant during exponential growth (Supplementary Fig. S8). The increased competition advantage when glutamate was added suggests that the *gltP* promoter mutations increase the expression of GltP, which in turn leads to an increased uptake of glutamate. Additionally, *gltP* promoter mutants, but not the wild-type, can grow on M9 minimal medium with 0.5% glutamate as carbon source.

arcA

In one of the evolved populations, two amino acid substitutions were found in *arcA*, the response regulator of the ArcAB two component regulatory system. ArcA represses numerous genes encoding enzymes in the TCA cycle and aerobic respiration, and acts as a switch to turn on anaerobic respiration (Gunsalus and Park, 1994; Kwon et al., 2000). No or a minor increase in fitness and exponential growth rate was detected for the re-constituted *arcA*(His40Pro) and *arcA*(Phe79Leu) mutations except for a positive effect for the *arcA*(His40Pro) mutation in combination with the otherwise apparently neutral *gltP*(-118delC) (4% fitness increase; Fig. 5A). An explanation for the observation could be that in a mutant that up-regulates *gltP*, down-regulation of *arcA* would be beneficial since this in turn would lead to an up-regulation of the pathways needed to convert *e.g.* glutamic acid into CO_2 and energy (the TCA cycle and aerobic respiration). *arcA* and *arcB* mutations have previously been found in a wide range of selections, including *E. coli* evolved in minimal medium supplemented with glucose and acetate (Herron and Doebeli, 2013; Le Gac and Doebeli, 2010), minimal medium with limiting glucose (Barrick et al., 2009), oxygen rich LB (Puentes-Téllez et al., 2014), as well as in *S. enterica* grown in Muller Hinton II medium (Hjort et al., 2016).

trkA/*trkH* and *sapF*/*sapD*

trkA/*trkH* and *sapF*/*sapD* have been designated as potassium transport genes where *sapF*/*sapD* confers ATP dependence to *trkH* (Harms et al., 2001; Schlosser et al., 1995). In addition, *sapF*/*sapD* have also been described as being part of an ABC transporter for cationic antimicrobial peptides (Shelton et al., 2011). The mutations *sapF*(Arg158Ser), *sapF*(Ser184Leu), *sapD*(Gly235Ser), and *trkH*(Pro94Ser) were re-constituted and tested for relative fitness and exponential growth (Fig. 5A). Out of the single mutants, only the *sapF* mutations conferred a measurable fitness enhancement in competition, and either no or a negative effect was detected on relative growth rates. However, *trkH* increased the fitness of the *gltP* mutant and could hence have been selected as a second mutation. When LB was supplemented with 2.5 mM KCl, a fitness decrease of the *trkH* mutant was seen but a corresponding decrease in fitness of the *sapF* and *sapD* mutants was not detected (Supplementary Fig. S7). We thus conclude that the *trkH* mutation is likely to increase the uptake of K^+ (LB contains no added K^+ and the bacteria probably starve for this ion). When K^+ was added to the medium, an increased K^+ uptake appeared deleterious.

rpoS and *rpoA*

In *E. coli* evolved in LB, three mutations were found in the RNA-polymerase subunits *rpoA* (alpha), *rpoS* (RpoS; *rpoA*[Asn294Lys] and *rpoS*[Ile95Phe and Ile128Asn]). Mutations in RNA polymerase subunits have repeatedly been found as adaptations during experimental evolution to a large variety of selective pressures and can for example direct the cells into faster metabolism (Conrad et. al 2010; Maharjan et al. 2012).

rpoS mutations were additionally found in both *E. coli* and *S. enterica* evolved in M9 glucose and it has been suggested that evolved *rpoS* mutations often reduce the affinity of RpoS to the RNA-polymerase (Conrad et al., 2009; Ferenci, 2005; Maharjan et al., 2012; Saxer et al., 2014). This results in decreased competition between RpoS and Sigma 70 to the RNA polymerase, and is thought to be beneficial under constant conditions with little stress.

The *rpoA*(Asn294His) mutation was previously also found in a study by Puentes-Téllez et al. (2014) where *E. coli* MC1000 was evolved in LB. This position is known to be important for αCTD–promoter DNA contacts (Lee et al., 2000) and substitutions with alanine at position 294 result in decreased CRP dependent transcription from the CC(-61.5) promoter and *metH* (Fritsch et al., 2000; Savery et al., 2002).

fim

See “MH – *E. coli*”

LB – *S. enterica*

Flagellar genes

Previous studies have shown that loss of flagella production constitutes around 3.2% fitness advantage in LB, which corresponds well to the estimated total energy expenditure of flagella (4.5%; Koskiniemi et al. 2012). Our results coincide with this observation as the Δ*flhE-D* which lacked flagella had a 4.1% benefit over an isogenic wild-type in competition experiments (Supplementary Fig. S7B). In cultures with rapid medium mixing, flagella and chemotaxis are not needed, and loss of these has been suggested to allow the cells to re-distribute a considerable amount of amino acids and ATP from building the flagellum and turning the flagellar motor to other processes (Edwards et al., 2002; Koskiniemi et al., 2012).

Furthermore, Stoebel et al. (2008) presented evidence that the rather high cost (~10%) of unnecessary expression of the *E. coli lac* operon was in the process of expression itself, *e.g.* through tying up RNA polymerase and ribosomes in expressing the *lac* genes instead of other genes. Considering the very high abundance of flagellin (FliC) in flagellated cells (>20,000 copies per flagellum; Minamino and Namba 2004), costs related to expression would be enough to explain the benefit of losing the flagellum.

Why then, were loss of flagella beneficial for *S. enterica* (in LB and MH medium) but not for *E. coli* grown in the same media? Many bacteria, including *E. coli* K12, express flagella

only when resources are limiting, as a means to find more nutrients. In contrast, *S. enterica* are motile when resources are plentiful, probably because motility is required for host colonization (Koirala et al., 2014). Specifically, the proportion of motile cells is tuned by nutrient availability, so that at high concentrations of yeast extract the majority of the cells are motile. As expected, motility assays in semisolid LB plates with our ancestral *S. enterica* and *E. coli* strains (Fig. S9) revealed differences in the level of motility: *S. enterica* was highly motile, quickly spreading out radially from the site of inoculation. *E. coli* on the other hand was less motile, only spreading slowly from the edge of the growing colony, probably as a response to local nutrient depletion.

As a result of this difference, the *E. coli* strain is probably mostly non-motile when grown in shaking LB cultures, while the *S. enterica* strain is highly motile. Thus, due to differences in regulation, expression of flagella and motility can confer a substantial cost for *S. enterica* growing in LB, and this cost is the likely cause for selection for loss of motility.

barA

The BarA-UvrY two-component regulatory system senses the metabolic state and activates the expression of many metabolic genes. It has been postulated that formate and acetate, end products of glucose metabolism, activate BarA-UvrY (Chavez et al., 2010). *barA* mutations have been suggested to lock the cells in a gluconeogenetic state (Pernestig et al., 2003; Suzuki et al., 2002), and would only be beneficial in conditions when no exogenous glucose is present. In agreement, when 0.2% glucose was added to the medium, the wild-type *barA* was advantageous over Δ*barA*, suggesting that the Δ*barA* mutant promotes utilization of the end products of glucose degradation which can enter the TCA cycle.

treB

treB encodes the trehalose specific IIBC PTS transporter component and is a pseudogene in the vast majority of all sequenced *S. typhimurium* genomes (all contain the same frameshift mutation, the deletion of an A in a stretch of 7 A:s starting at nucleotide position 538, counted from the first nucleotide in the start codon). It is unclear whether this is selected or just a historical accident. However, the reversion, or pseudo-reversion, happens frequently in our wild-type *S. enterica* in LB by insertion of a nucleotide at the site of the original frameshift mutation or in the neighboring codons. The *treB*$^{-}$ ancestor produces tiny, slow-growing colonies on M9 plates with trehalose as sole carbon source (Supplementary Table S5; Fig. S11), indicating intrinsic frameshift suppression or, alternatively, that trehalose can enter the cell through some other, less efficient route. Revertants and pseudo-revertants (*treB*$^{+}$) grow to visible colonies overnight.

As expected, supplementing the LB medium with 0.2% trehalose enhanced the positive effect of *treB*$^{+}$ mutants in head-to-head competition experiments with the *treB*$^{-}$ un-evolved ancestor from about 4 to 8% (Supplementary Fig. S7B). Comparing the growth curves of the wild-type (*treB*$^{-}$) strain to the *treB*$^{+}$ mutant with and without added trehalose in the medium further showed that the enhanced positive effect of *treB*$^{+}$ in the presence of trehalose was at a late state in the growth curve (Supplementary Fig. S7D).

Although LB generally is assumed to contain no sugar (Sezonov et al., 2007), significant amounts of glucose, galactose and trehalose have been reported, with the latter being the most abundant (Baev et al., 2006; Ferreira et al., 1997; Hanko and Rohrer, 2000, 2004). We suggest that the selective advantage of the *treB*$^{+}$ revertants in LB was due to the improved utilization of trehalose as carbon and/or energy source.

MH – *E. coli*

rph-pyrE

E. coli K12 carries the frameshift mutation *rph-1* (in the gene encoding RNase PH), resulting in polar effects on expression of *pyrE* and, consequently, partial pyrimidine auxotrophy (Pyr^{-}; Bonekamp et al. 1984; Jensen 1993). Our mutants compensated for this by (i) reverting the frameshift in *rph* or (ii), putatively, by increasing read-through of the transcriptional attenuator between the genes and thereby rescuing the leaky Pyr^{-}. With an *rph*$^{+}$ genotype, the translation

of RNase PH is terminated 10 bp upstream of the transcriptional terminator, whereas in *E. coli* K12, this occurs 36 bp upstream of the terminator. With the 82 bp deletion, the translation of RNase PH is instead terminated just after the native transcription terminator, leading to increased levels of *pyrE*. This exact mutation was previously found by *e.g.* Conrad et al. (2009) and Herring et al. (2006) after evolving *E. coli* K12 MG1655 in lactate minimal medium. They concluded that the deletion relieves the pyrimidine biosynthesis defect in MG1655.

fim and *flu*

The phase variable expression of fimbriae in *E. coli* is catalyzed by the site-specific recombinases FimE and FimB. FimE catalyzes the inversion of a 314 bp DNA element containing the *fim* promoter, from the ON (towards *fimA*) to the OFF (away from *fimA*) orientation (Holden et al., 2007), whereas FimB catalyzes inversion in both directions. Loss of FimE leads to a lower rate of switching from ON to OFF, resulting in a higher fraction of fimbriated cells. In our laboratory, wild-type *E. coli* strain (DA5438) the *fim* promoter is in the OFF orientation, while in all the MH adapted strains it is in the ON orientation.

In competition experiments the *fimE*::IS*5* mutant had an advantage over the ancestral parent whereas our constructed Δ*fimBEAICDFGH* mutant was deleterious (1.081 ± 0.015 and 0.833 ± 0.006, respectively; Fig. 5C). Thus, the IS*5* insertion switched the phase variable promoter in front of *fimA* into the ON orientation, indicating that fimbriation was beneficial in MH.

The MH evolved *E. coli* populations also acquired mutations in the coding sequence and promoter of the gene *flu* (*agn43*), which is involved in cell aggregation and late stages of biofilm formation, and is under phase variable control (Beloin et al., 2008). We see no obvious explanation for why mutations affecting *flu* are beneficial in MH, but it is suggestive that both *flu* and *fim* mutations that are involved in different stages of biofilm formation and cell aggregation were selected in this medium. Curiously, expression of *flu* is repressed in cells that express fimbriae (Beloin et al., 2008).

MH – *S. enterica*

malT and *malQ*

MalT is the transcriptional activator of the maltose regulon, which is important for catabolism of maltose and maltodextrins (Ferenci, 1980; Nanavati et al., 2005). However, our wild-type *S. enterica* (DA6192) carries three mutations in the *mal*-regulon which lead to a *mal*$^-$ phenotype (*malQ*[Leu96Arg], *malP*[Ile458fs; Δ37 bp], and *malT*[-46+A]). It cannot utilize maltose, but can grow slowly on minimal medium with maltodextrin (Supplementary Table S5). Loss of MalQ makes cells unable to utilize maltose, while loss of MalP results in cells unable to use maltodextrin (Park et al., 2011). Thus, our wild-type is phenotypically *malQ*$^-$, *malP*$^+$ (despite the frameshift mutation in *malP*). Furthermore, a *malQ* mutant accumulates maltose and maltotriose in the presence of maltodextrins. MalT is activated by the binding of maltotriose, and activates transcription of the *mal* regulon. In a MalQ$^-$, MalP$^+$ mutant, this could cause a costly positive feedback loop that turns on the *mal* regulon fully even at low concentrations of maltodextrins, and make the cells unable to tune down expression as MalQ is unable to further degrade maltotriose. Similarly, expression of the *E. coli lac* operon is costly when induced by the non-metabolizable lactose analog IPTG (Stoebel et al., 2008).

MH does not contain any added maltose or maltodextrin, but does contain starch, which could possibly decompose into maltodextrins and maltose during medium preparation. The *malT* mutants were thus probably selected as compensation for the preexisting mutations in our wild-type strain (unique for DA6192). The enhanced relative fitness of the Δ*malT* mutant when maltose was added to the medium further supports this hypothesis. Interestingly, in one of the evolved populations we found in addition to the *malT*(Gln855*) mutation that was present in 76% of the sequence reads, 16% of the sequence reads contained the mutation *malQ*(Arg96Cys). As stated above, our wild-type carries the *malQ*(Leu96Arg) mutation in the same codon and does not grow on maltose, but grows, albeit poorly, on maltodextrin (phenotypically Mal$^-$, Mdx$^{(+)}$; Supplementary Tables S2 and S5). This population contained

clones that were phenotypically Mal^+, Mdx^+, confirmed by local sequencing to be *malQ*(Arg96Cys) *malT*$^+$, as well as clones that were phenotypically Mal^-, Mdx^-, confirmed to carry the *malT* mutation but not the *malQ* pseudoreversion. Hence, it appears that our *S. enterica* could increase growth in MH by either (i) loss of the activator of the *mal*-regulon and thereby gain fitness because of a reduced cost of expression of the non-functional *mal* genes or (ii) through pseudo-reversion of the *malQ*(Leu96Arg) mutation which leads to a Mal^+ phenotype.

M9 glycerol – *E. coli*

rph-pyrE

See "MH – *E. coli*"

glpK

Mutations in the glycerol kinase *glpK* were found in both *E. coli* and *S. enterica* evolved in M9 glycerol (Tables S1 – S2) and mutations in two of the positions (Val8 and Arg34) were shared between the species. GlpK catalyzes the first step in glycerol catabolism and is a known target for mutations in *E. coli* B and K12 evolved in M9 glycerol (Applebee et al., 2011; Herring et al., 2006; Le Gac et al., 2013). Glycerol phosphorylation catalyzed by GlpK is the first and rate limiting step for glycerol utilization (Zwaig et al., 1970). Glycerol feeds into glycolysis (and gluconeogenesis) as dihydroxyacetone-phosphate. Fructose-1,6-bisphosphate (FBP) from gluconeogenesis acts as a non-competitive allosteric inhibitor of glycerol kinase, reducing the flux from glycerol into carbon and energy metabolism. Thus, mutations that remove this allosteric inhibition allows for better utilization of glycerol as sole carbon and energy source (Applebee et al., 2011; Herring et al., 2006). Several mutations in the FBP binding loop (positions 230-237; IGGKGGTR) have previously been found in *E. coli* GlpK (Anderson et al., 2007; Applebee et al., 2011) and were also found in this study. Among these, G231D was found to abolish the binding of FBP and increase enzymatic activity (Anderson et al., 2007) and we find it very likely that the mutations in the FBP binding loop selected in this study have similar effects.

Other mutations, V62L, D73V, A66T and S59W, in the dimer-dimer interface disrupts tetramerization and have also been found to result in insensitivity towards inhibition by FBP (Applebee et al., 2011; Bystrom et al., 1999; Liu et al., 1994). Similarly, we isolated mutations A66V and S58R, as well as a mutation in position 34 which are also part of the dimer-dimer interface in the crystal structure of GlpK (Anderson et al., 2007). In addition, we isolated mutations in positions 8, 19, 157, 189, and 325, which are not particularly close to the dimer-dimer interface, FBP binding loop or any other known functional site. However, mutations identical to ours at positions 19 and 325 have been isolated previously in glycerol adapted *E. coli* (Applebee et al., 2011).

rhs

The 'recombination hotspot' loci (*rhs*) has been found to confer a means for contact dependent growth inhibition through a set of interchangeable toxin-antitoxin-like modules. Recombination within and between *rhs* loci enable switching between toxin types, which enable cells to inhibit their relatives (Koskiniemi et al., 2013). The *rhsB-rhsA* duplications were not analyzed further.

M9 glycerol – *S. enterica*

glpK

See "M9 glycerol – *E. coli*"

nadR

nadR mutations were adaptive in *S. enterica* during growth in M9 glycerol. The gene encodes a protein with three activities. (i) the N-terminal part of the protein binds DNA and represses

expression of genes involved in NAD synthesis. (ii) the central regulatory domain binds nicotinamide mononucleotide, which acts as a co-repressor, and (iii), the C terminus has an enzymatic function that is part of a scavenging pathway that makes NAD+ from precursors taken up from the medium (Grose et al., 2005). In M9 glycerol no NAD+ precursors are present, which is why *de novo* NAD+ synthesis is needed. Thus, it is likely that de-repression of genes involved in *de novo* NAD+ synthesis was beneficial in this medium.

In line with this we found that Δ*nadR* was beneficial. Some of the evolved mutations were within the central domain (Gln134fs and Trp157Arg). Mutations within this domain (*e.g.* the mutation Gln134*) have previously been shown to de-repress genes involved in NAD+ synthesis (Grose et al., 2005; Zhu and Roth, 1991). Other mutations were in the C-terminal domain (Leu251Phe, Gly290Asp, Ala317Glu, and Glu374*), which are not directly involved in transcription repression. It is, however, not unlikely that these mutations affect the structure, expression or stability of the NadR protein, leading to reduced repression.

nadR has repeatedly been found mutated during experimental evolution of *E. coli* B grown in minimal medium supplemented with glucose or glycerol (Le Gac et al., 2013) or glucose and acetate (Herron and Doebeli, 2013; Le Gac et al., 2008). Herron and Doebeli (2013) found an identical *nadR* mutation as we did (Gly290Asp) in mutants from two different evolution experiments.

M9 glucose – *E. coli*

rpoB and *rpoS*

See "*E. coli* – LB"

rph-pyrE

See "*E. coli* – MH"

proQ

ProQ was recently identified as a major small-RNA binding protein in *S. enterica,* binding to more than 400 transcripts and stabilizing many of them (Smirnov et al., 2016). We tested three different mutations (Asp92fs, Leu17Gln, and Glu130*), which had similar effects on relative fitness and relative exponential growth rates. Previously, probable loss of function mutations in *proQ* have been found in lactate minimal medium (Conrad et al., 2009).

M9 glucose – *S. enterica*

pykF

In *S. enterica*, all four lineages had acquired deletions, frameshift mutations or amino acid substitutions in *pykF* (pyruvate kinase I). A constructed Δ*pykF* mutant showed an increase in both competitive fitness and exponential growth rate (Fig. 6D). Deletions of *pykF* have repeatedly been found in *E. coli* adapted to glucose minimal medium and LB (Barrick and Lenski, 2013; Blaby et al., 2012; Woods et al., 2006) and it has been suggested that the mutations increase glucose uptake by reducing the conversion of phosphoenolpyruvate (PEP) to pyruvate (Woods et al., 2006). See also main text ("Adaptation to M9 glucose")

fes/fep and *iroN*

See Supplementary Table S6.

References

Aguilar, C., Escalante, A., Flores, N., de Anda, R., Riveros-McKay, F., Gosset, G., et al. (2012). Genetic changes during a laboratory adaptive evolution process that allowed fast growth in glucose to an Escherichia coli strain lacking the major glucose transport system. BMC Genomics 13, 385. doi:10.1186/1471-2164-13-385.

Anderson, M. J., DeLaBarre, B., Raghunathan, A., Palsson, B. O., Brunger, A. T., and Quake, S. R. (2007). Crystal structure of a hyperactive Escherichia coli glycerol kinase mutant Gly230Asp obtained using microfluidic crystallization devices. Biochemistry 46, 5722–5731. doi:10.1021/bi700096p.

Applebee, M. K., Joyce, A. R., Conrad, T. M., Pettigrew, D. W., and Palsson, B. (2011). Functional and metabolic effects of adaptive glycerol kinase (GLPK) mutants in Escherichia coli. J. Biol. Chem. 286, 23150–23159. doi:10.1074/jbc.M110.195305.

Baev, M. V., Baev, D., Jansco Radek, A., and Campbell, J. W. (2006). Growth of Escherichia coli MG1655 on LB medium: Monitoring utilization of amino acids, peptides, and nucleotides with transcriptional microarrays. Appl. Microbiol. Biotechnol. 71, 317–322. doi:10.1007/s00253-005-0310-5.

Barrick, J. E., and Lenski, R. E. (2013). Genome dynamics during experimental evolution. Nat. Rev. Genet. 14, 827–39. doi:10.1038/nrg3564.

Barrick, J. E., Yu, D. S., Yoon, S. H., Jeong, H., Oh, T. K., Schneider, D., et al. (2009). Genome evolution and adaptation in a long-term experiment with: Escherichia coli : Abstract : Nature. Nature 461, 1243–1247. Available at: http://www.nature.com/nature/journal/v461/n7268/abs/nature08480.html%5Cnpapers2://publication/doi/10.1038/nature08480.

Beloin, C., Roux, A., and Ghigo, J. M. (2008). Escherichia coli biofilms. Curr. Top. Microbiol. Immunol. 322, 249–289. doi:10.1007/978-3-540-75418-3_12.

Blaby, I. K., Lyons, B. J., Wroclawska-Hughes, E., Phillips, G. C. F., Pyle, T. P., Chamberlin, S. G., et al. (2012). Experimental evolution of a facultative thermophile from a mesophilic ancestor. Appl. Environ. Microbiol. 78, 144–155. doi:10.1128/AEM.05773-11.

Bonekamp, F., Clemmesen, K., Karlstrom, O., and Jensen, K. F. (1984). Mechanism of UTP-modulated attenuation at the pyrE gene of Escherichia. EMBO J 3, 2857–2861.

Brandis, G., and Hughes, D. (2013). Genetic characterization of compensatory evolution in strains carrying rpoB Ser531Leu, the rifampicin resistance mutation most frequently found in clinical isolates. J. Antimicrob. Chemother. 68, 2493–2497. doi:10.1093/jac/dkt224.

Brandis, G., Pietsch, F., Alemayehu, R., and Hughes, D. (2015). Comprehensive phenotypic characterization of rifampicin resistance mutations in Salmonella provides insight into the evolution of resistance in Mycobacterium tuberculosis. J. Antimicrob. Chemother. 70, 680–685. doi:10.1093/jac/dku434.

Bystrom, C. E., Pettigrew, D. W., Branchaud, B. P., O'Brien, P., and Remington, S. J. (1999). Crystal structures of Escherichia coli glycerol kinase variant S58-->W in complex with nonhydrolyzable ATP analogues reveal a putative active conformation of the enzyme as a result of domain motion. Biochemistry 38, 3508–3518. doi:10.1021/bi982460z.

Charusanti, P., Conrad, T. M., Knight, E. M., Venkataraman, K., Fong, N. L., Xie, B., et al. (2010). Genetic basis of growth adaptation of Escherichia coli after deletion of pgi, a major metabolic gene. PLoS Genet. 6, e1001186. doi:10.1371/journal.pgen.1001186.

Chavez, R. G., Alvarez, A. F., Romeo, T., and Georgellis, D. (2010). The physiological stimulus for the BarA sensor kinase. J. Bacteriol. 192, 2009–2012.

doi:10.1128/JB.01685-09.

Conrad, T. M., Frazier, M., Joyce, A. R., Cho, B.-K., Knight, E. M., Lewis, N. E., et al. (2010). RNA polymerase mutants found through adaptive evolution reprogram Escherichia coli for optimal growth in minimal media. Proc. Natl. Acad. Sci. U. S. A. 107, 20500–20505. doi:10.1073/pnas.0911253107.

Conrad, T. M., Joyce, A. R., Applebee, M. K., Barrett, C. L., Xie, B., Gao, Y., et al. (2009). Whole-genome resequencing of Escherichia coli K-12 MG1655 undergoing short-term laboratory evolution in lactate minimal media reveals flexible selection of adaptive mutations. Genome Biol. 10, R118. doi:10.1186/gb-2009-10-10-r118.

Edwards, R. J., Sockett, R. E., and Brookfield, J. F. Y. (2002). A simple method for genome-wide screening for advantageous insertions of mobile DNAs in Escherichia coli. Curr. Biol. 12, 863–867. doi:10.1016/S0960-9822(02)00837-0.

Ferenci, T. (1980). The Recognition of Maltodextrins by Escherichia coli. Eur. J. Biochem. 108, 631–636. doi:10.1111/j.1432-1033.1980.tb04758.x.

Ferenci, T. (2005). Maintaining a healthy SPANC balance through regulatory and mutational adaptation. Mol. Microbiol. 57, 1–8. doi:10.1111/j.1365-2958.2005.04649.x.

Ferreira, J. C., Paschoalin, V. M. F., Panek, A. D., and Trugo, L. C. (1997). Comparison of three different methods for trehalose determination in yeast extracts. Food Chem. 60, 251–254. doi:10.1016/S0308-8146(96)00330-5.

Fritsch, P. S., Urbanowski, M. L., and Stauffer, G. V. (2000). Role of the RNA polymerase ?? subunits in MetR-dependent activation of metE and metH: Important residues in the C-terminal domain and orientation requirements within RNA polymerase. J. Bacteriol. 182, 5539–5550. doi:10.1128/JB.182.19.5539-5550.2000.

Grose, J. H., Bergthorsson, U., and Roth, J. R. (2005). Regulation of NAD synthesis by the trifunctional NadR protein of Salmonella enterica. J. Bacteriol. 187, 2774–2782. doi:10.1128/JB.187.8.2774-2782.2005.

Gunsalus, R. P., and Park, S. J. (1994). Aerobic-anaerobic gene regulation in Escherichia coli: control by the ArcAB and Fnr regulons. Res. Microbiol. 145, 437–450. doi:10.1016/0923-2508(94)90092-2.

Hanko, V. P., and Rohrer, J. S. (2000). Determination of carbohydrates, sugar alcohols, and glycols in cell cultures and fermentation broths using high-performance anion-exchange chromatography with pulsed amperometric detection. Anal. Biochem. 283, 192–199. doi:10.1006/abio.2000.4653.

Hanko, V. P., and Rohrer, J. S. (2004). Determination of amino acids in cell culture and fermentation broth media using anion-exchange chromatography with integrated pulsed amperometric detection. Anal. Biochem. 324, 29–38. doi:10.1016/j.ab.2003.09.028.

Harms, C., Domoto, Y., Celik, C., Rahe, E., Stumpe, S., Schmid, R., et al. (2001). Identification of the ABC protein SapD as the subunit that confers ATP dependence to the K+-uptake systems TrkH and TrKG from Escherichia coli K-12. Microbiology 147, 2991–3003. doi:10.1099/00221287-147-11-2991.

Hayashi, K., Morooka, N., Yamamoto, Y., Fujita, K., Isono, K., Choi, S., et al. (2006). Highly accurate genome sequences of Escherichia coli K-12 strains MG1655 and W3110. Mol. Syst. Biol. 2, 2006.0007. doi:10.1038/msb4100049.

Herring, C. D., Raghunathan, A., Honisch, C., Patel, T., Applebee, M. K., Joyce, A. R., et al. (2006). Comparative genome sequencing of Escherichia coli allows observation of bacterial evolution on a laboratory timescale. Nat. Genet. 38, 1406–1412. doi:10.1038/ng1906.

Herron, M. D., and Doebeli, M. (2013). Parallel Evolutionary Dynamics of Adaptive

Diversification in Escherichia coli. PLoS Biol. 11, e1001490. doi:10.1371/journal.pbio.1001490.

Hjort, K., Nicoloff, H., and Andersson, D. I. (2016). Unstable tandem gene amplification generates heteroresistance (variation in resistance within a population) to colistin in Salmonella enterica. Mol. Microbiol. 0, 1–16. doi:10.1111/mmi.13459.

Holden, N., Blomfield, I. C., Uhlin, B. E., Totsika, M., Kulasekara, D. H., and Gally, D. L. (2007). Comparative analysis of FimB and FimE recombinase activity. Microbiology 153, 4138–4149. doi:10.1099/mic.0.2007/010363-0.

Jensen, K. F. (1993). The Escherichia coli K-12 “wild types” W3110 and MG1655 have an rph frameshift mutation that leads to pyrimidine starvation due to low pyrE expression levels. J. Bacteriol. 175, 3401–3407.

Knöppel, A., Näsvall, J., and Andersson, D. I. (2017). Evolution of antibiotic resistance without antibiotic exposure. Antimicrob. Agents Chemother., AAC.01495-17. doi:10.1128/AAC.01495-17.

Koirala, S., Mears, P., Sim, M., Golding, I., Chemla, Y. R., Aldridge, P. D., et al. (2014). A nutrient-tunable bistable switch controls motility in Salmonella enterica serovar Typhimurium. MBio 5, e01611-14. doi:10.1128/MBIO.01611-14.

Koskiniemi, S., Lamoureux, J. G., Nikolakakis, K. C., t’Kint de Roodenbeke, C., Kaplan, M. D., Low, D. A., et al. (2013). Rhs proteins from diverse bacteria mediate intercellular competition. Proc. Natl. Acad. Sci. 110, 7032–7037. doi:10.1073/pnas.1300627110.

Koskiniemi, S., Sun, S., Berg, O. G., and Andersson, D. I. (2012). Selection-driven gene loss in bacteria. PLoS Genet. 8, e1002787. doi:10.1371/journal.pgen.1002787.

Kwon, O., Georgellis, D., and Lin, E. C. C. (2000). Phosphorelay as the sole physiological route of signal transmission by the Arc two-component system of Escherichia coli. J. Bacteriol. 182, 3858–3862. doi:10.1128/JB.182.13.3858-3862.2000.

Le Gac, M., Brazas, M. D., Bertrand, M., Tyerman, J. G., Spencer, C. C., Hancock, R. E. W., et al. (2008). Metabolic changes associated with adaptive diversification in Escherichia coli. Genetics 178, 1049–1060. doi:10.1534/genetics.107.082040.

Le Gac, M., Cooper, T. F., Cruveiller, S., Médigue, C., and Schneider, D. (2013). Evolutionary history and genetic parallelism affect correlated responses to evolution. Mol. Ecol. 22, 3292–3303. doi:10.1111/mec.12312.

Le Gac, M., and Doebeli, M. (2010). Epistasis and frequency dependence influence the fitness of an adaptive mutation in a diversifying lineage. Mol. Ecol. 19, 2430–2438. doi:10.1111/j.1365-294X.2010.04664.x.

Lee, D. J., Wing, H. J., Savery, N. J., and Busby, S. J. (2000). Analysis of interactions between Activating Region 1 of Escherichia coli FNR protein and the C-terminal domain of the RNA polymerase alpha subunit: use of alanine scanning and suppression genetics. Mol. Microbiol. 37, 1032–1040. Available at: http://www.ncbi.nlm.nih.gov/pubmed/10972822.

Liu, W. Z., Faber, R., Feese, M., Remington, S. J., and Pettigrew, D. W. (1994). Escherichia coli glycerol kinase: role of a tetramer interface in regulation by fructose 1,6-bisphosphate and phosphotransferase system regulatory protein IIIglc. Biochemistry 33, 10120–6. Available at: http://www.ncbi.nlm.nih.gov/pubmed/8060980.

Maharjan, R. P., Ferenci, T., Reeves, P. R., Li, Y., Liu, B., and Wang, L. (2012). The multiplicity of divergence mechanisms in a single evolving population. Genome Biol 13, R41. doi:10.1186/gb-2012-13-6-r41.

Minamino, T., and Namba, K. (2004). Self-assembly and type III protein export of the bacterial flagellum. J. Mol. Microbiol. Biotechnol. 7, 5–17. doi:10.1159/000077865.

Mokrousov, I., Otten, T., Vyshnevskiy, B., and Narvskaya, O. (2003). Allele-specific rpoB PCR assays for detection of rifampin-resistant Mycobacterium tuberculosis in sputum smears. Antimicrob. Agents Chemother. 47, 2231–2235. Available at: http://www.ncbi.nlm.nih.gov/pubmed/12821473.

Nanavati, D. M., Nguyen, T. N., and Noll, K. M. (2005). Substrate Specificities and Expression Patterns Reflect the Evolutionary Divergence of Maltose ABC Transporters in Thermotoga maritima. J. Bacteriol. 187, 2002–2009.

Näsvall, J., Knöppel, A., and Andersson, D. I. (2016). Duplication-Insertion Recombineering: a fast and scar-free method for efficient transfer of multiple mutations in bacteria. Nucleic Acids Res. 45, e33. doi:10.1093/nar/gkw1078.

Park, J. T., Shim, J. H., Tran, P. L., Hong, I. H., Yong, H. U., Oktavina, E. F., et al. (2011). Role of maltose enzymes in glycogen synthesis by Escherichia coli. J. Bacteriol. 193, 2517–2526. doi:10.1128/JB.01238-10.

Pernestig, A. K., Georgellis, D., Romeo, T., Suzuki, K., Tomenius, H., Normark, S., et al. (2003). The Escherichia coli BarA-UvrY two-component system is needed for efficient switching between glycolytic and gluconeogenic carbon sources. J. Bacteriol. 185, 843–853. doi:10.1128/JB.185.3.843-853.2003.

Puentes-Téllez, P. E., Hansen, M. A., Sørensen, S. J., and van Elsas, J. D. (2013). Adaptation and heterogeneity of escherichia coli MC1000 growing in complex environments. Appl. Environ. Microbiol. 79, 1008–1017. doi:10.1128/AEM.02920-12.

Puentes-Téllez, P. E., Kovács, Á. T., Kuipers, O. P., and van Elsas, J. D. (2014). Comparative genomics and transcriptomics analysis of experimentally evolved Escherichia coliMC1000 in complex environments. Environ. Microbiol. 16, 856–870. doi:10.1111/1462-2920.12239.

Savery, N. J., Lloyd, G. S., Busby, S. J. W., Thomas, M. S., Ebright, R. H., and Gourse, R. L. (2002). Determinants of the C-terminal domain of the Escherichia coli RNA polymerase ?? subunit important for transcription at class I cyclic AMP receptor protein-dependent promoters. J. Bacteriol. 184, 2273–2280. doi:10.1128/JB.184.8.2273-2280.2002.

Saxer, G., Krepps, M. D., Merkley, E. D., Ansong, C., Deatherage Kaiser, B. L., Valovska, M. T., et al. (2014). Mutations in Global Regulators Lead to Metabolic Selection during Adaptation to Complex Environments. PLoS Genet. 10, e1004872.

Schlosser, A., Meldorf, M., Stumpe, S., Bakker, E. P., and Epstein, W. (1995). TrkH and its homolog, TrkG, determine the specificity and kinetics of cation transport by the Trk system of Escherichia coli. J. Bacteriol. 177, 1908–1910.

Sezonov, G., Joseleau-Petit, D., and D'Ari, R. (2007). Escherichia coli physiology in Luria-Bertani broth. J. Bacteriol. 189, 8746–8749. doi:10.1128/JB.01368-07.

Shelton, C. L., Raffel, F. K., Beatty, W. L., Johnson, S. M., and Mason, K. M. (2011). Sap transporter mediated import and subsequent degradation of antimicrobial peptides in Haemophilus. PLoS Pathog. 7, e1002360. doi:10.1371/journal.ppat.1002360.

Smirnov, A., Förstner, K. U., Holmqvist, E., Otto, A., Günster, R., Becher, D., et al. (2016). Grad-seq guides the discovery of ProQ as a major small RNA-binding protein. Proc. Natl. Acad. Sci. 113, 11591–11596. doi:10.1073/pnas.1609981113.

Spencer, C. C., Tyerman, J., Bertrand, M., and Doebeli, M. (2008). Adaptation increases the likelihood of diversification in an experimental bacterial lineage. Proc. Natl. Acad. Sci. U. S. A. 105, 1585–9. doi:10.1073/pnas.0708504105.

Stoebel, D. M., Dean, A. M., and Dykhuizen, D. E. (2008). The cost of expression of Escherichia coli lac operon proteins is in the process, not in the products. Genetics 178,

1653–1660. doi:10.1534/genetics.107.085399.

Suzuki, K., Wang, X., Weilbacher, T., Pernestig, A. K., Melefors, Öjar, Georgellis, D., et al. (2002). Regulatory circuitry of the CsrA/CsrB and BarA/UvrY systems of Escherichia coli. J. Bacteriol. 184, 5130–5140. doi:10.1128/JB.184.18.5130-5140.2002.

Telenti, A., Imboden, P., Marchesi, F., Lowrie, D., Cole, S., Colston, M. J., et al. (1993). Detection of rifampicin-resistance mutations in Mycobacterium tuberculosis. Lancet (London, England) 341, 647–650. Available at: http://www.ncbi.nlm.nih.gov/pubmed/8095569.

Tenaillon, O., Barrick, J. E., Ribeck, N., Deatherage, D. E., Blanchard, J. L., Dasgupta, A., et al. (2016). Tempo and mode of genome evolution in a 50,000-generation experiment. Nature 536, 165–70. doi:10.1038/nature18959.

Tenaillon, O., Rodriguez-Verdugo, A., Gaut, R. L., McDonald, P., Bennett, A. F., Long, A. D., et al. (2012). The Molecular Diversity of Adaptive Convergence. Science (80-.). 335, 457–461. doi: 10.1126/science.1212986.

Wallecha, A., Oreh, H., van der Woude, M. W., and deHaseth, P. L. (2014). Control of gene expression at a bacterial leader RNA, the agn43 gene encoding outer membrane protein Ag43 of Escherichia coli. J. Bacteriol. 196, 2728–2735. doi:10.1128/JB.01680-14.

Wang, L., Spira, B., Zhou, Z., Feng, L., Maharjan, R. P., Li, X., et al. (2010). Divergence involving global regulatory gene mutations in an Escherichia coli population evolving under phosphate limitation. Genome Biol. Evol. 2, 478–487. doi:10.1093/gbe/evq035.

Wichelhaus, T. A., Schäfer, V., Brade, V., and Böddinghaus, B. (1999). Molecular characterization of rpoB mutations conferring cross-resistance to rifamycins on methicillin-resistant Staphylococcus aureus. Antimicrob. Agents Chemother. 43, 2813–2816. Available at: http://www.ncbi.nlm.nih.gov/pubmed/10543773.

Woods, R., Schneider, D., Winkworth, C. L., Riley, M. A., and Lenski, R. E. (2006). Tests of parallel molecular evolution in a long-term experiment with Escherichia coli. Proc. Natl. Acad. Sci. U. S. A. 103, 9107–9112. doi:10.1073/pnas.0602917103.

Yamagishi, A., Tanimoto, T., Suzuki, T., and Oshima, T. (1996). Pyrimidine biosynthesis genes (pyrE and pyrF) of an extreme thermophile, Thermus thermophilus. Appl. Environ. Microbiol. 62, 2191–2194.

Zhu, N., and Roth, J. R. (1991). The nadI region of Salmonella typhimurium encodes a bifunctional regulatory protein. J. Bacteriol. 173, 1302–1310.

Zorraquino, V., Kim, M., Rai, N., and Tagkopoulos, I. (2017). The Genetic and Transcriptional Basis of Short and Long Term Adaptation across Multiple Stresses in Escherichia coli. Mol. Biol. Evol. 34, 707–717.

Zwaig, N., Kistler, W. S., and Lin, E. C. (1970). Glycerol kinase, the pacemaker for the dissimilation of glycerol in Escherichia coli. J. Bacteriol. 102, 753–759.
